# Comparative transcriptome analysis reveals the patterns of gene expression in different venison cuts of sika deer (*Cervus nippon*)

**DOI:** 10.5713/ab.25.0044

**Published:** 2025-05-12

**Authors:** Lan Tang, Qianghui Wang, Haihua Xing, Yukai Ma, Zihui Sun, Tao Zhu, Heping Li

**Affiliations:** 1 College of Wildlife and Protected Area, Northeast Forestry University, Harbin, China

**Keywords:** Gene Expression, Meat Quality, Sika Deer, Transcriptome, Venison

## Abstract

**Objective:**

Venison (deer meat) is increasingly favored by consumers for its safety, nutrition, flavor, and natural qualities. However, venison cuts in different regions of the carcass have different meat quality due to their distinct physiological function. To investigate the molecular basis of these variations, RNA-seq was used to compare gene expression pattern across different cuts of venison.

**Methods:**

In this study, we performed a transcriptomic analysis based on RNA-seq data of 72 cuts from 6 different body parts of sika deer (*Cervus nippon*, n = 12), with the aim of understanding the genetic factors affecting the differences in venison quality, providing important references for further deer breeding.

**Results:**

We identified 139,111 expressed genes, including 79 region-specific genes enriched in pathways crucial for meat texture, tenderness, and nutrition. Differential gene expression analysis revealed significant variations among venison cuts, especially between the longissimus dorsi and trapezius cuts, highlighting roles in metal ion transport, organic acid biosynthesis, and glycolysis/gluconeogenesis. Additionally, fatty acid metabolism genes showed stable expression, while muscle fiber structure genes varied, affecting tenderness and juiciness.

**Conclusion:**

Our findings provide insights into the genetic factors influencing venison quality, offering a foundation for future breeding strategies to enhance meat quality.

## INTRODUCTION

Venison, a premium meat product, is an excellent source of protein, amino acids, and polyunsaturated fatty acids. It also boasts a low cholesterol content, making it a valuable animal food resource [1–4]. However, there are significant differences in the quality of venison from different cuts, such as tenderness, meat color, pressurized water loss rate, intramuscular fat levels, and the pH_24h_ [4]. Jin et al. found that the pH values for different cuts of sika deer meat range from 5.49 to 5.78, the tenderness ranges between 31.71 N and 68.53 N, the intramuscular fat content ranges from 0.66% to 4.97%, and the water-holding capacity ranges from 71.00% to 73.78% [4]. These factors have a significant impact on the nutrition and flavor of venison. Additional, China once classified deer as protected animals and prohibited their use for meat. It wasn’t until 2020 that they were reclassified as specialty livestock (main including sika deer and red deer), allowing venison to be served on dining tables again. This policy change has led to significant transformations in China’s deer industry, with many farms beginning to breed deer for meat production. The vast market potential has continuously stimulated the development of the venison industry [5,6]. Nonetheless, because the venison industry started relatively late, research on venison quality lags far behind that of beef and lamb. There is particularly a lack of study on the quality differences between various cuts of venison and the underlying mechanisms driving these differences. This has significantly hindered the development of the deer industry and public awareness of venison, making it difficult to select and breed deer for meat production. Therefore, our research into the mechanisms driving differences in meat quality is extremely urgent.

High-throughput transcriptomics offers a sensitive and precise method for analysis. It allows researchers to explore transcriptional landscapes comprehensively. This approach is particularly useful for studying biological systems in detail [7]. RNA-Seq technology was widely applied in research on meat quality in livestock and poultry [8–11]. For instance, Fonseca et al explored global gene expression differences in various beef cuts, and they found several potential candidate genes related to meat tenderness [8]. Yu et al investigated the muscle-specific molecular differences between the tenderloin and longissimus lumborum in cattle during the early postmortem period. Their findings revealed 65 differentially expressed genes (DEGs) associated with energy production and conversion, transcription, and oxidative phosphorylation [9]. Yu et al utilized transcriptomic and metabolomic analyses to reveal the regulatory mechanisms of intramuscular fat content in beef cattle [11]. Meng et al compared the meat quality of Simmental and Chinese native cattle in the longissimus lumborum. They identified two key signaling pathways related to meat quality — endoplasmic reticulum and adipocytokine signaling — and highlighted several candidate genes, including *LEPR*, *HSPA12A*, and *CAPN1* [10]. Those showed that transcriptomic studies have yielded substantial findings in beef research, laying a solid foundation for beef studies and cattle breeding. However, transcriptomic research on venison remains limited, with existing studies primarily concentrating on anatomical and nutritional aspects. For example, Serrano et al conducted a comparative study on the differences between hunted and farmed red deer venison. Their findings indicated that venison from farmed deer exhibited higher protein content, lower shear force, and reduced levels of certain components. In contrast, venison from hunted deer showed a higher concentration of total, essential, and non-essential amino acids [12]. Additionally, previous research has classified venison into three types: red, dark, and saddle. Red venison, the tenderloin, is the most tender of all cuts. Dark venison is found in the hind legs and neck, while saddle venison is located between the loin and the shank. Each of these cuts has its own unique flavor, texture, and cooking methods [1,12–14]. Therefore, with the ongoing development of the deer farming industry, investigating the regulatory mechanisms underlying differences in meat quality has become particularly important.

To investigate the molecular mechanisms underlying the differences in the formation of various venison cuts, we conducted a comparative transcriptomic analysis on six venison cuts from adult sika deer. We first evaluated the expression levels of candidate genes in 72 muscle samples across six different regions. Region-specific genes (RSGs) related to the distinct meat qualities of the venison cuts were identified, and regulatory transcription factors (TFs) for these RSGs were detected. Subsequently, we assessed the correlation between gene expression levels in different venison cuts. Several important candidate genes with diverse expression patterns among venison cuts were identified, and these genes were differentially expressed, contributing to meat quality with essential nutritional components.

## MATERIALS AND METHODS

All experimental designs and animal handling were approved by the Institutional Animal Care and Use Committee of Northeast Forestry University, China (2022049).

### Sample collection

**Figure 1:**
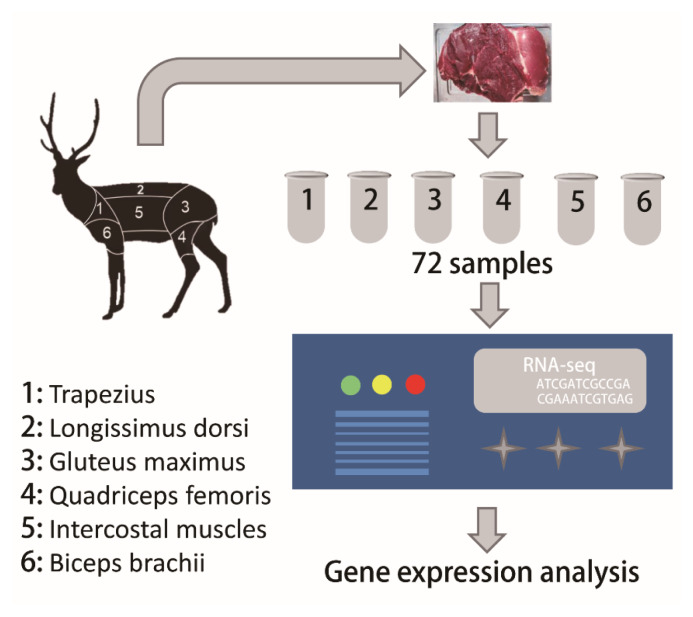
Global framework of the present study. We used 72 venison cuts from six male and six female adult sika deer to study the expression specificity patterns through multifaceted analyses (including gene expression, region specific expression, differentially expressed gene analysis, fatty acid content related gene and muscle fiber structure related gene expression analysis).

A total of 72 venison samples from six male and six female adult sika deer were collected from Shuangyang Deer Quality Breed Breeding (Changchun, China). Before slaughtering, 12 sika deer were fattened under the same feeding and management conditions until they were 2 years old. Then, these sika deer were transferred to Jilin Province Changchun Shuangyang District Shilu Deer Industry Group for slaughter. Tissue samples were collected with the approved by the Institutional Animal Care and Use Committee of Northeast Forestry University, China (2022049). A total of six types of venison cuts, including longissimus dorsi (LD), intercostal muscles (IM), trapezius (T), biceps brachii (BB), gluteus maximus (GM), and quadriceps femoris (QF) were collected and saved in RNAlater and snap-frozen in liquid nitrogen (Figure 1).

### RNA extraction and RNA sequencing

Total RNA was extracted from each sample using the Trizol method, subjected to quality control using the NanoDrop® 2000 (Thermo, Waltham, MA, USA), and treated with DNase I (RNase-free) according to the manufacturer’s instructions. mRNA libraries were prepared following the TruSeq Stranded library protocols with 5 μg of total RNA. An insert size of 150 bp was selected with AMPure XP beads and used for polymerase chain reaction (PCR) enrichment for library construction. To ensure library quality, PCR products were purified using AMPure XP beads and assessed on the Agilent Bioanalyzer 2100 system. Finally, raw sequence data were generated for each sample using the Illumina NovaSeq 6000 platform.

### Data quality control, mapping and gene expression analysis

The sequencing raw data were trimmed for high-quality reads using FASTP software (v0.20.1) with default parameters [15]. Then, clean data for each sample were mapped to the reference genome (GCA_040085125.1) using HISAT2 (v2.0.4) [16]. HTseq [17] was used to calculate read count, and finally, gene expression levels were calculated as fragments per kilobase of transcript per million mapped fragments (FPKM) were estimated according to the formula “FPKM = (number of reads in gene × 10^9^)/(number of all reads in genes × the gene length)”.

### Region-specific genes detection

The method was applied to identify RSGs [18,19]. Briefly, RSGs were defined by three criteria: 1) The FPKM value of the candidate gene in one type of venison cut was more than three times that of others; 2) The FPKM value in one type of venison cut was greater than 50% of the average expression level in all others; 3) The expression level was within the top 25% of all genes in each venison cut. Hierarchical clustering of RSGs was performed using the Pheatmap R package. Functional annotation and enrichment analysis of RSGs were conducted using the Database for Annotation, Visualization, and Integrated Discovery. RSGs in six types of venison cuts were aligned with AnimalTFDB v2.0 to identify TF expression in the venison cuts [20]. Additionally, the protein-protein interaction (PPI) network of RSGs in different venison cuts was generated using the String database (https://string-db.org/), and visualized using Cytoscape (v.3.7.1) [21].

### Different expression gene analysis

To investigate the potential impact of differential gene expression on meat quality in various venison cuts, we conducted 15 groups of comparative analyses, primarily including BB vs GM, IM vs GM, IM vs BB, T vs GM, T vs BB, T vs IM, QF vs GM, QF vs BB, QF vs IM, QF vs T, LD vs GM, LD vs BB, LD vs IM, LD vs T, and LD vs QF. DESeq2 (v1.28.1) was employed to identify DEGs based on the read count values of various cuts in the previous steps [22]. DEGs were identified with a threshold of false discovery rate<0.05 and |log2(fold change)|>1. Gene ontology (GO) enrichment analyses for each comparison group were conducted using EnrichPipeline (based on GO database) [23]. KOBAS (v3.0) was used to Kyoto encyclopedia of genes and genomes (KEGG) enrichment analysis for DEGs [24]. Detailly, we first annotate the genes with KEGG annotation by referring to the KEGG Orthology terms for *Bos taurus* (bta) in the KOBAS database, and then perform enrichment analysis using the KOBAS tool.

### Fatty acid content and muscle fiber structure related gene expression analysis

To analyze the intramuscular fat content and muscle fiber structure in different venison cuts, fatty acid biosynthesis, degradation, elongation, and muscle fiber structure were given priority. By reviewing the literature and gene function annotations, we compared the genes involved in the fatty acid biosynthesis, degradation, and elongation pathways across different venison cuts. Additionally, we referenced the key genes identified by Zhang et al in their beef studies related to muscle fiber structure formation [19]. We compiled and compared the expression levels of these genes in various venison cuts. After quantifying the expression levels, ggplot2 package was used to visualize the gene expression levels related to fatty acid biosynthesis, degradation, elongation, and muscle fiber structure.

## RESULTS

### RNA sequencing and transcriptomes analysis

**Figure 2:**
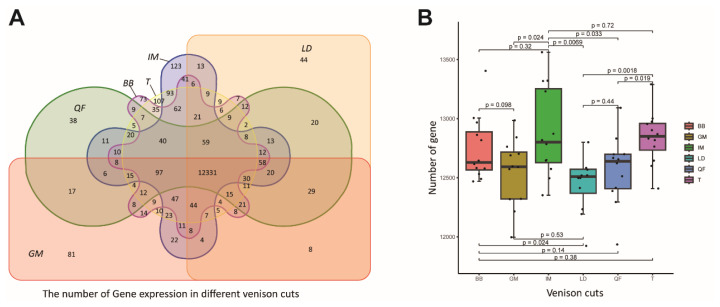
Expressed gene numbers, shared and unique genes in different venison cuts. (A) Number of shared and unique expressed genes identified in six types of venison cuts. (B) The number of expressed genes in six types of venison cuts. There are significant differences in the number of genes between LD and T, QF and T, IM and QF, IM and LD, IM and GM, and BB and LD (p<0.05). LD, longissimus dorsi; IM, intercostal muscles; T, trapezius. BB, biceps brachii; QF, quadriceps femoris; GM, gluteus maximus.

To study pattern in gene expression among venison cuts, we performed RNA-Seq analysis on 72 muscle samples taken from 6 adult male and 6 adult female sika deer (Figure 1). A total of 4,147,735,364 raw reads (~622.17 Gb) were generated from RNA sequencing of venison cut samples (Supplement 1). Specifically, a total of 68.95, 69.19, 69.19, 69.32, 70.47, and 67.66 million reads were obtained from the LD, IM, T, BB, GM, and QF cuts, respectively. In total, 96.97% of the raw reads passed the quality control, and an average of 94.37% (ranging from 92.19% to 96.2%) clean reads were mapped to the sika deer reference genome (Supplement 2) [25]. In this study, we found that the number of expressed genes (FPKM≥1) was highest in the IM (12,885±383) and lowest in the LD (12,475±272). The number of expressed genes in the GM and QF was similar, with 12,548±291 and 12,567±284 genes, respectively. The number of expressed genes in the IM was significantly higher than that in the LD, QF, and GM (p<0.05) (Figure 2B). These results are similar to previous findings in beef studies [19]. In addition, 12,331 genes were commonly expressed in six venison cuts, and 44, 81, 38, 73, 107, and 123 genes were uniquely expressed in LD, GM, QF, BB, T, and IM, respectively (Figure 2A). To eliminate the influence of confounding factors at the experimental level, we retained genes with FPKM values greater than 1 in the 12 biological replicate samples. A total of 13,911 genes were obtained for the downstream analyses.

### Region-specific expression patterns analysis

**Figure 3:**
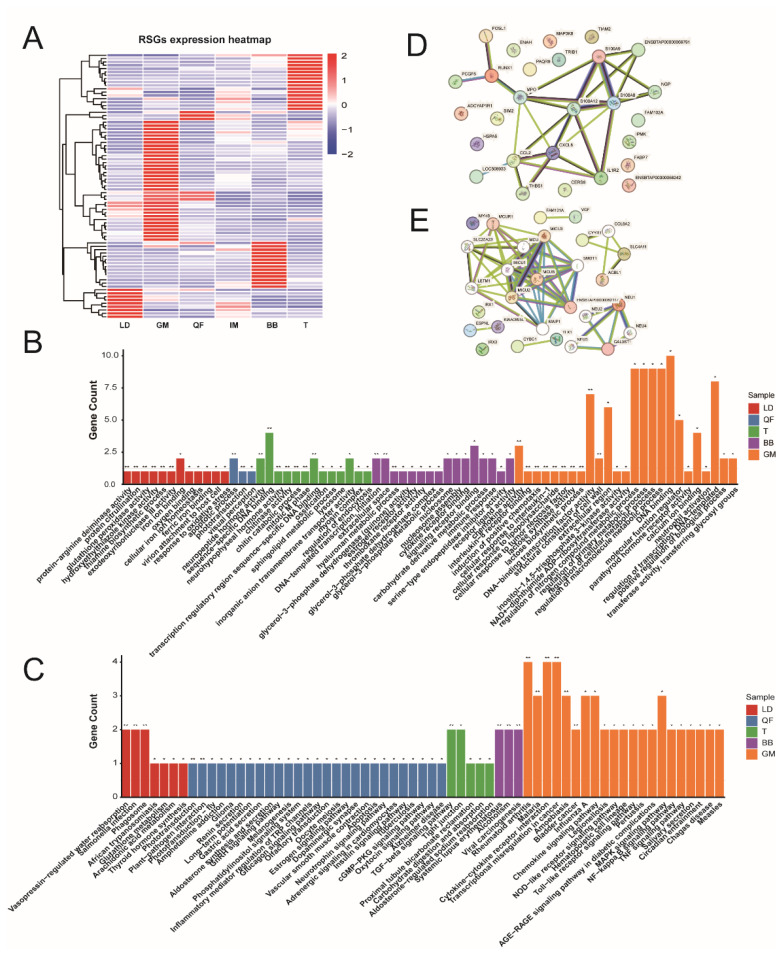
RSGs identification and functional analysis. (A) The heatmap of RSGs expression. (B) GO Enrichment analysis results of RSGs, the significance is indicated as * p<0.05, ** p<0.01, the bar color is indicated as different samples. (C) KEGG enrichment analysis results of RSGs, the significance is indicated as * p<0.05, ** p<0.01, the bar color is indicated as different samples. (D) Protein to protein interaction in LD. (E) Protein to protein interaction in GM. RSGs, region-specific genes; GO, gene ontology; KEGG, Kyoto encyclopedia of genes and genomes; LD, longissimus dorsi.

To identify the region-specific expression genes in different venison cuts, based on the similar detection methods as described by a previous study [18], we identified a total of 79 region-specific genes (RSGs) among 13,911 genes from six different types of venison cuts (Figure 3A). Among the various cuts, the GM cut exhibited the highest number of RSGs (n = 34, including *MAP3K8*, *IGLL5*, *CCL2*, *HSPA6*, *S100A8*, *S100A9*, *ADCYAP1R1*, *FAM102A*, *IL1R2*, *TSP1*, *SIM2, RUNX1*, *PAQR9*, *IPMK*, *KGP1*, *PCGF5*, *TRIB1*, *CHM4C*, *ENAH*, *CERS6*, *IGHG3*, *F181A*, *ATS9*, *LYSCN*, *HXC11*, *AGRF1*, *TPD53*, *FABP7*, *AMC2*, *IL8*, *FOSL1*, *TIAM2*), followed by the T cut with 17 RSGs (including *NEU1*, *S4A1*, *IRX3*, *ESPNL*, *CLD1*, *TLX1*, *ATNG*, *CH3L1*, *HXD3*, *IRX1*, *MCUB*, *OSTP*, *ACHG*, *VGF*, *MYH8*), the BB cut with 14 RSGs (including *EPAB2*, *SG2B2*, *FEL1A*, *SG2B2*, *CRBB2*, *ITIH1*, *BSL1*, *MYLK4*, *H4*, *PTN5*, *COX19*), the LD cut with 9 RSGs (including *DC1I*, *GPX2*, *HBB*, *SLC15A1*, *LEMD1*, *PADI2*, *SIM1*) and the QF cut with 5 RSGs (including *CIDEA*, *PRVA*, *CIDEA*, *HXC11*) (Figure 3A). To comprehensively explore the functions of the RSGs across different cuts, we observed that the RSGs of GM are significantly associated with various biological processes (BP) and pathways, including chemokine activity (GO:0008009, p = 0.00015), interleukin-8 receptor binding (GO:0005153, p = 0.0016), regulation of primary metabolic processes (GO:0080090, p = 0.014), calcium ion binding (GO:0005509, p = 0.032), structural components of the cell wall (GO:0005199, p = 0.0085), as well as the tumor necrosis factor signaling pathway (p = 0.034), NF-kappa B signaling pathway (p = 0.032), and MAPK signaling pathway (p = 0.029). In contrast, the RSGs of T are predominantly implicated in neuropeptide hormone activity (GO:0005184, p = 0.00038), chitinase activity (GO:0004568, p = 0.0048), and the myosin complex (GO:0016459, p = 0.033). They also participate in the transforming growth factor (TGF)-beta signaling pathway (p = 0.0056), tight junction formation (p = 0.023), carbohydrate digestion and absorption (p = 0.048), and aldosterone-regulated sodium reabsorption (p = 0.049). The RSGs of BB are primarily involved in the glycerol-3-phosphate metabolic process (GO:0006072, p = 0.018), carbohydrate derivative metabolic processes (GO:1901135, p = 0.03), and serine-type endopeptidase inhibitor activity (GO:0004867, p = 0.048). Meanwhile, the RSGs of LD are mainly engaged in protein-arginine deiminase activity (GO:0004668, p = 0.0021), glutathione peroxidase activity (GO:0004602, p = 0.003), iron ion binding (GO:0005506, p = 0.01), cellular iron ion homeostasis (GO:0006879, p = 0.029), the thiamine biosynthetic process (GO:0009228, p = 0.0047), vasopressin-regulated water reabsorption (p = 0.00034), and glutathione metabolism (p = 0.034). In the cuts of QF, the RSGs are involved in apoptotic process (GO:0006915, p = 0.00073), cGMP-PKG signaling pathway (p = 0.047), Glucagon signaling pathway (p = 0.03), Gastric acid secretion (p = 0.02), and Aldosterone synthesis and secretion (p = 0.027) (Figures 3B, 3C).

TFs play a regulatory role in gene expression [26,27]. To investigate whether the RSGs in different venison cuts are regulated by TFs, 6 TFs (including *IRX1*, *IRX3*, *TLX1*, *HXD3*, *RUNX1*, and *Fosl1*) were found in RSGs. These TFs are primarily found in the LD and GM cuts. Based on PPIs, we constructed interaction networks for the RSG proteins in the LD and GM cuts (Figures 3D, 3E).

### Differential expression gene analysis in venison cuts

**Figure 4:**
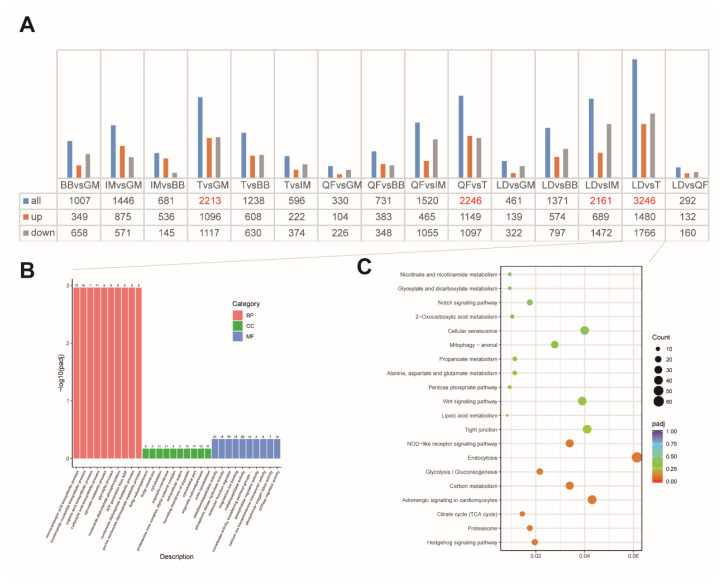
Identification of DEGs. (A) The number of up-regulated and down-regulated DEGs identified in each group, the red bar represents the number of upregulated genes, gray bar represents the number of down-regulated genes, and blue represents the total number of DEGs in the corresponding tissue. (B) GO enrichment results of DEGs between LD and T. (C) KEGG enrichment results of DEGs between LD and T. BB, biceps brachii; GM, gluteus maximus; IM, intercostal muscles; T, trapezius; QF, quadriceps femoris; LD, longissimus dorsi; DEGs, differentially expressed genes; GO, gene ontology; KEGG, Kyoto encyclopedia of genes and genomes.

The diverse quality among different venison cuts implies the potential difference of genetic basis under selection during the breed formation [12,13]. Here, we evaluated transcriptome changes using RNA-seq on the six primary cuts (i.e., LD, BB, IM, T, QF, GM) of venison and investigated the DEGs (Figure 1). We observed that the number of DEGs among venison cuts ranges from 292 in LD vs QF (132 upregulated and 160 downregulated genes) to 3,246 in LD vs T (1,480 upregulated and 1,766 downregulated genes). For other compare venison cuts, we found 1,007, 1,446, 681, 2,213, 1,238, 596, 330, 731, 1,520, 2,246, 461, 1,271, and 2,161 DEGs in BB vs GM, IM vs GM, IM vs BB, T vs GM, T vs BB, T vs IM, QF vs GM, QF vs BB, QF vs T, LD vs GM, LD vs BB, and LD vs IM, respectively (Figure 4A).

To assess the functional contribution of DEGs for venison quality, we performed functional enrichment analysis. Firstly, we focused on LD vs T, which exhibited the greatest number of DEGs, which were enriched in molecular function (MF) GO terms including metalloendopeptidase activity (GO: 0004222, p = 0.0032), magnesium ion binding (GO:0000287, p = 0.007), MF regulator (GO:0098772, p = 0.0055), calcium ion transmembrane transporter activity (GO:0015085, p = 0.0164), BP GO terms including monocarboxylic acid biosynthetic process (GO:0072330, p = 1.17e-5) nicotinamide nucleotide biosynthetic process (GO:0019359, p = 2.68e-5), organic acid biosynthetic process (GO:0016053, p = 2.86e-5), glycolytic process (GO:0006096, p = 3.65e-5), carboxylic acid biosynthetic process (GO:0046394, p = 2.86e-5), pyruvate biosynthetic process (GO:0042866, p = 3.65e-5), ATP generation from ADP (GO:0006757, p = 2.86e-5), and cellular component GO terms including Golgi subcompartment (GO: 0098791, p = 0.0167), cytoskeleton (GO:0005856, p = 0.0573) (Figure 4B; Supplement 3). KEGG enrichment showed that DEGs of LD vs T were involved in Hedgehog signaling pathway (p = 0.00012), glycolysis/gluconeogenesis (p = 0.0017), lipoic acid metabolism (p = 0.015), and alanine, aspartate and glutamate metabolism (p = 0.036) (Figure 4C; Supplement 4). In addition, we found that DEGs of other venison cuts enriched in several critical functions/signaling pathways, such as vitamin digestion and absorption (p<0.05), vasopressin-regulated water reabsorption (p<0.05), protein digestion and absorption (p<0.05), TGF-beta signaling pathway (p<0.05), Wnt signaling pathway (p<0.05), AMPK signaling pathway (p<0.05), MAPK signaling pathway (p<0.05), regulation of lipolysis in adipocytes (p<0.05), starch and sucrose metabolism (p<0.05), glycolysis/gluconeogenesis (p<0.05), immune response (GO:0006955, p<0.05), monocarboxylic acid biosynthetic process (GO:0072330, p<0.05), lipid metabolic process (GO:0006629, p<0.05), organic acid biosynthetic process (GO:0016053, p<0.05), vitamin binding (GO:0019842, p<0.05), amino acid activation (GO:0043038, p<0.05), and thyroid hormone synthesis (p<0.05) (Supplements 5–32). These signaling or functions pathways provide crucial molecular evidence for the nutritional value formation of venison from different anatomical locations.

### Muscle fiber structure and fatty acid content of venison cuts

**Figure 5:**
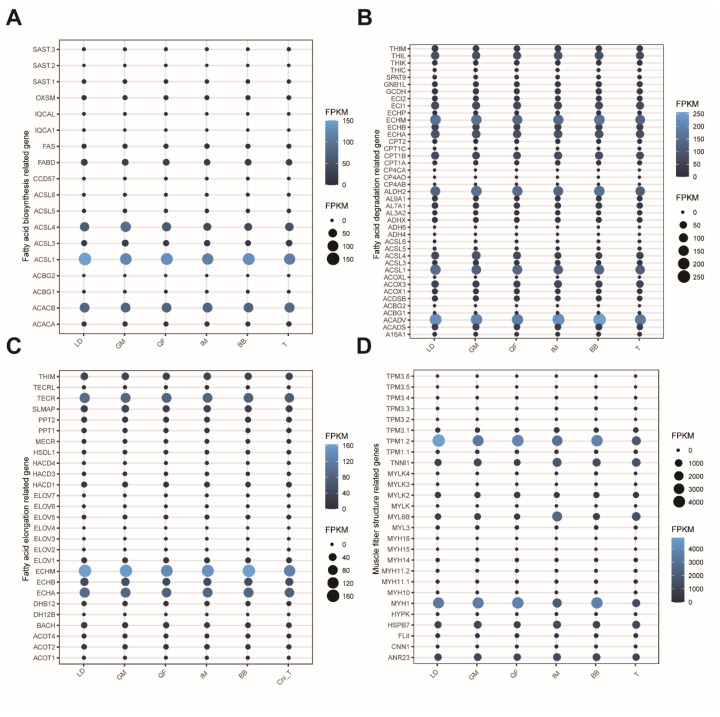
Muscle fiber structure and fatty acid candidate gene expression profile. The x-axis represents six types of venison cuts, namely the longissimus dorsi, intercostal muscles, trapezius, biceps brachii, gluteus maximus, and quadriceps femoris. The y-axis represents the expression level of candidate genes. (A, B, C) Fatty acid biosynthesis, degradation, elongation related genes expression levels in six venison cuts. (D) Muscle fiber structure related genes expression levels in six venison cuts. FPKM, fragments per kilobase of transcript per million mapped fragments; LD, longissimus dorsi; GM, gluteus maximus; QF, quadriceps femoris; IM, intercostal muscles; BB, biceps brachii; T, trapezius.

Fatty acid content and muscle fiber structure are critical factors influencing meat quality and nutritional value [19,28–30]. To investigate these aspects in six different venison cuts, we specifically compared the expression levels of key genes involved in fatty acid biosynthesis, degradation, elongation, and muscle fiber structure (Figure 5). Our analysis revealed that genes related to fatty acid biosynthesis, including *SAST*, *OXSM*, *IQCAL*, *IQCA1*, *FAS*, *FABD*, *CCD57*, *ACSL1*, *ACSL3–6*, *ACBG1*, *ACBG2*, and *ACAC*, did not show significant differences in expression levels among the six venison cuts (Figure 5A). Similarly, genes associated with fatty acid degradation (e.g., *THIM*, *THIL*, *THIK*, *THIC*, *SPAT9*, *GNB1L*, *GCDH*, *ECI*, *ECHP*, *ECHM*, *ECHA*, *ECHB*, *CPT2*, *CPT1A*, *CPT1B*, *CPT1C*, *CP4CA*, *CP4AO*, *CP4AB*, *AL9A1*, *AL7A1*, *AL3A2*, *ADH*, *ACSL1*, *ACS3–6*, *ACOXL*, *ACOX1*, *ACOX3*, *ACDSB*, *ACBG2*, *ACBG1*, *ACADV*, *ACADS*, *A16A1*) and fatty acid elongation (e.g., *THIM*, *TECRL*, *TECR*, *SLMAP*, *PPT1*, *PPT2*, *MECR*, *HSDL1*, *HACD*, *ELOV*, *ECHM*, *ECHB*, *ECHA*, *DHB12*, *BACH*, *ACOT1*, *ACOT2*, *ACOT4*) did not exhibit significant differences in expression levels across the six venison cuts (Figures 5B, 5C).

In contrast, genes related to muscle fiber structure showed varying expression levels among different tissues. Specifically, *TPM1* and *MYH1* exhibited relatively lower expression levels in T and IM cut, while their expression levels were consistent in LD, BB, and QF cuts. Additionally, *MYL6B* and *TNNI1* were expressed at higher levels in IM cuts compared to other venison cuts. The expression levels of other muscle fiber structure-related genes were relatively similar across the different venison cuts (Figure 5D).

## DISCUSSION

We performed a comparative analysis of the expression pattern using RNA-seq technology in different muscle tissues from various venison cuts. Many previous studies have been applied to investigate the genetic basis of meat quality using different gene expressions [8–11]. In here, RNA-seq was applied to venison research for the first time. We collected 72 venison samples and obtained 622.17G of transcriptome data through sequencing for gene expression analysis. The data validity and mapping rate both exceeded 90% (Supplement 2), providing important assurance for the reliability and accuracy of the gene expression analysis.

The specific expression of genes is closely associated with physiological functions and life processes. Region-specific transcriptomic analyses in various livestock have provided valuable insights into the regulation of gene expression that underlies functional differences among different regions of the body [18,19]. In our study, we identified 79 RSGs among six different venison cuts. These RSGs are enriched in processes such as chitinase activity regulation of primary metabolic process, thiamine biosynthetic process, transcription regulatory region sequence-specific DNA binding, vasopressin-regulated water reabsorption, cGMP-PKG signaling pathway, and TGF-beta signaling pathway. Primary metabolic processes encompass the metabolism of proteins, fats, and carbohydrates, which directly influence the nutritional content and flavor of meat [31]. Therefore, regulating these primary metabolic processes can directly impact the tenderness, flavor, and freshness of venison. Additionally, thiamine, an important form of vitamin B1, plays a crucial role in energy metabolism and nerve function. The thiamine content in meat affects its nutritional value [32]. Previous studies have shown that the TGF-beta signaling pathway is involved in the formation and differentiation of muscle fibers and the generation of connective tissue [33]. By regulating the growth of muscle cells and the formation of connective tissue, the TGF-beta signaling pathway significantly impacts the hardness and texture of meat. In other words, the TGF-beta signaling pathway can substantially affect venison by influencing muscle fiber formation. The vasopressin helps maintain fluid balance by regulating water reabsorption in the kidneys [34]. This process significantly affects the overall hydration of the animal, which directly impacts the water content of its muscles, thereby noticeably affecting the juiciness and tenderness of the meat [34]. Overall, the specific expression of RSGs can significantly influence the tenderness, juiciness, and flavor of different venison cuts, which corresponds to the varying quality observed among different cuts of venison [2,35].

The genetic basis for the formation of differences in meat quality is due to differential gene expressions [36–38]. In here, we found that gene expression varies significantly in different cuts of venison, with the highest number of DEGs between LD and T (n = 3,246) (Figure 4). These genes are involved in functions or signaling pathways such as metal ion transport and binding, organic acid biosynthesis, amino acid metabolism, and glycolysis/gluconeogenesis (Figures 4B, 4C). The binding and transport of metal ions have significant effects on muscle contraction, metabolism, and physiological characteristics [39]. Magnesium ion is an important cofactor in protein synthesis, influencing the synthesis and degradation of muscle proteins, thus impacting muscle growth and meat quality [40]. Calcium ions play a crucial role in muscle contraction. An increase in calcium ion concentration within muscle cells promotes contraction, while a decrease leads to relaxation. Calcium ion transmembrane transport proteins directly influence muscle contraction ability and muscle quality by regulating the influx and efflux of calcium ions [39]. In addition, the organic acid biosynthetic process, and glycolysis/gluconeogenesis will significantly affect the pH value of meat. Previous studies indicated that the pH value of most meat pH values of 5.5 to 5.7 measured approximately 24 hours post slaughter (so called ultimate pH) are within the normal range, while values over 5.8 result in reduced shelf life, especially for vacuum-packaged meat [41]. Interestingly, our study found that multiple groups of DEGs were involved in the organic acid biosynthetic process, and glycolysis/gluconeogenesis, which provide important references for our subsequent selection and breeding of deer for venison.

Flavor, tenderness, and nutrient content are important characteristics of the quality of meat and are also what consumers focus on when selecting meat [1]. Fat acid content and muscle fiber structure are important factors influencing these characteristics. Our study found that there were no obvious differences in the expression of key genes involved in fatty acid synthesis, degradation, and elongation in different parts of venison, indicating that the fatty acid content across different venison cuts is relatively stable (Figures 5A–5C), which is consistent with previous research results [1,12–14]. Regarding the expression of key genes involved in muscle fiber structure formation, *TPM1*, *MYH1*, *TNNI1*, and *MYL6B* varied among different venison cuts (Figure 5D). *TPM1* is involved in muscle fiber transformation, and different types of muscle fibers affect meat quality differently [42]. *MYH1* and *MYL6B* are involved in muscle contraction and fast-twitch muscle fiber activity [43–45], which in turn affects the meat’s texture and taste. The expression of *TNNI1* is mainly in slow-twitch oxidative muscle fibers, which have an important impact on meat tenderness and water-holding capacity [46–47]. *TPM1* and *MYH1* have lower expression levels in the IM and T muscles, which is consistent with the characteristics of these muscle cuts. Meanwhile, *MYL6B* and *TNNI1* were expressed at higher levels in IM cuts compared to other venison cuts. Compared to more active muscles like LD, GM, BB, and QF, the muscle fibers in IM and T are relatively lower, resulting in more tenderness and juiciness. Therefore, the study of muscle fiber structure is extremely important for the breeding of deer for meat production, as it can significantly improve the taste and texture of venison. Unlike beef and lamb, low-fat content is an important characteristic of venison, and studies have found that there may be no significant difference in fat content across different cuts. Therefore, low-fat percentage may not need to be a primary focus in the selection and breeding of deer for meat.

## SUPPLEMENTARY MATERIAL

**Supplement 1**. Statistics summary of sequencing data for transcriptomes.

**Supplement 2**. Statistics of sample mapping rate.

**Supplement 3**. The KEGG enrichment results of DEGs between LD and T.

**Supplement 4**. The GO enrichment results of DEGs between LD and T.

**Supplement 5**. The KEGG enrichment results of DEGs between LD and QF.

**Supplement 6**. The GO enrichment results of DEGs between LD and QF.

**Supplement 7**. The KEGG enrichment results of DEGs between LD and IM.

**Supplement 8**. The GO enrichment results of DEGs between LD and IM.

**Supplement 9**. The KEGG enrichment results of DEGs between LD and BB.

**Supplement 10**. The GO enrichment results of DEGs between LD and BB.

**Supplement 11**. The KEGG enrichment results of DEGs between LD and GM.

**Supplement 12**. The GO enrichment results of DEGs between LD and GM.

**Supplement 13**. The KEGG enrichment results of DEGs between QF and T.

**Supplement 14**. The GO enrichment results of DEGs between QF and T.

**Supplement 15**. The KEGG enrichment results of DEGs between QF and IM.

**Supplement 16**. The GO enrichment results of DEGs between QF and IM.

**Supplement 17**. The KEGG enrichment results of DEGs between QF and BB.

**Supplement 18**. The KEGG enrichment results of DEGs between QF and BB.

**Supplement 19**. The KEGG enrichment results of DEGs between QF and GM.

**Supplement 20**. The GO enrichment results of DEGs between QF and GM.

**Supplement 21**. The KEGG enrichment results of DEGs between T and IM.

**Supplement 22**. The GO enrichment results of DEGs between T and IM.

**Supplement 23**. The KEGG enrichment results of DEGs between T and BB.

**Supplement 24**. The GO enrichment results of DEGs between T and BB.

**Supplement 25**. The KEGG enrichment results of DEGs between IM and BB.

**Supplement 26**. The GO enrichment results of DEGs between IM and BB.

**Supplement 27**. The KEGG enrichment results of DEGs between IM and GM.

**Supplement 28**. The GO enrichment results of DEGs between IM and GM.

**Supplement 29**. The KEGG enrichment results of DEGs between BB and GM.

**Supplement 30**. The GO enrichment results of DEGs between BB and GM.

**Supplement 31**. The KEGG enrichment results of DEGs between T and GM.

**Supplement 32**. The GO enrichment results of DEGs between T and GM.
