# Supplementary material for: Comparative transcriptome analysis reveals the patterns of gene expression in different venison cuts of sika deer (Cervus nippon)
Source: Anim Biosci. 2025 May 12;38(11):2324–35. doi: 10.5713/ab.25.0044 (PMC12580950; doi:10.5713/ab.25.0044)
Supplement: Supplementary file 1 [file ab-25-0044-supplementary-1.pdf]

Supplement 1. Statistics summary of sequencing data for transcriptomes

Sample	raw_reads	raw_bases	clean_reads	clean_bases	error_rate	Q20	Q30	GC_percent
HG_1_LD	61441550	9.22G	60011646	9.0G	0.01	98.48	95.42	52.98
HG_1_GM	55881496	8.38G	54233864	8.14G	0.01	98.67	96.14	51.73
HG_1_QF	59581010	8.94G	58230416	8.73G	0.01	98.9	96.89	51.95
HG_1_IM	56302884	8.45G	54917540	8.24G	0.01	98.44	95.33	53.16
HG_1_BB	56968078	8.55G	55511236	8.33G	0.01	98.51	95.45	49.64
HG_1_T	54289690	8.14G	53150158	7.97G	0.01	98.55	95.57	46.89
HG_2_LD	61959212	9.29G	60346730	9.05G	0.01	98.59	95.74	51.99
HG_2_GM	55240000	8.29G	53654196	8.05G	0.01	98.46	95.36	52.61
HG_2_QF	53988218	8.1G	52590594	7.89G	0.01	98.61	96.03	50.13
HG_2_IM	55001768	8.25G	53278602	7.99G	0.01	98.47	95.44	51.89
HG_2_BB	58819444	8.82G	56287764	8.44G	0.01	98.84	96.83	52.53
HG_2_T	52422300	7.86G	51758450	7.76G	0.01	98.25	94.7	51.36
HG_3_LD	55036910	8.26G	53806282	8.07G	0.01	98.5	95.65	51.51
HG_3_GM	60785572	9.12G	59406174	8.91G	0.01	98.73	96.48	52.29
HG_3_QF	53925372	8.09G	53031692	7.95G	0.01	98.19	94.5	49.81
HG_3_IM	53723578	8.06G	53043642	7.96G	0.01	98.88	96.82	49.29
HG_3_BB	54703646	8.21G	53510328	8.03G	0.01	98.38	95.15	51.74
HG_3_T	54187830	8.13G	53030914	7.95G	0.01	98.14	94.42	53.44
HG_4_LD	54903944	8.24G	54281960	8.14G	0.01	98.68	96.01	48.59
HG_4_GM	54361212	8.15G	53061102	7.96G	0.01	98.49	95.35	51.57
HG_4_QF	54577224	8.19G	53236098	7.99G	0.01	98.57	95.84	52.15
HG_4_IM	54362300	8.15G	53222206	7.98G	0.01	98.39	95.09	52
HG_4_BB	55646240	8.35G	54386622	8.16G	0.01	98.59	95.97	52.97
HG_4_T	54742362	8.21G	53567042	8.04G	0.01	98.6	95.84	51.41
HG_5_LD	55568104	8.34G	54784558	8.22G	0.01	98.42	95.18	50.5
HG_5_GM	59196782	8.88G	57613508	8.64G	0.01	98.46	95.32	52.37
HG_5_QF	67104238	10.07G	66448088	9.97G	0.01	99.05	97.23	47.87
HG_5_IM	54587368	8.19G	53681646	8.05G	0.01	98.47	95.3	50.5
HG_5_BB	53896486	8.08G	52733568	7.91G	0.01	98.29	94.8	52.95
HG_5_T	63690370	9.55G	62332538	9.35G	0.01	98.74	96.41	52.47
HG_6_LD	53427144	8.01G	52750464	7.91G	0.01	98.43	95.18	51.21
HG_6_GM	56951196	8.54G	55282792	8.29G	0.01	98.16	94.51	52.79
HG_6_QF	55074340	8.26G	53872502	8.08G	0.01	98.42	95.19	52.68
HG_6_IM	62429460	9.36G	61242100	9.19G	0.01	98.89	96.81	53
HG_6_BB	62131994	9.32G	61288756	9.19G	0.01	98.83	96.63	51.1
HG_6_T	58349568	8.75G	56637520	8.5G	0.01	98.51	95.46	53.17
HM_1_LD	56439912	8.47G	55018806	8.25G	0.01	98.55	95.81	52.78
HM_1_GM	67287832	10.09G	66455434	9.97G	0.01	98.96	97.01	49.01
HM_1_QF	55603186	8.34G	54026308	8.1G	0.01	98.54	95.73	52.68
HM_1_IM	66160936	9.92G	64869216	9.73G	0.01	98.16	94.46	52.66
HM_1_BB	62759002	9.41G	61398572	9.21G	0.01	98.8	96.58	52.66

HM_1_T	55844364	8.38G	54147172	8.12G	0.01	98.29	94.84	52.58
HM_2_LD	58138564	8.72G	57050040	8.56G	0.01	98.38	95.06	52
HM_2_GM	54570940	8.19G	53491810	8.02G	0.01	98.52	95.51	53.55
HM_2_QF	61601778	9.24G	60524858	9.08G	0.01	98.5	95.42	52.77
HM_2_IM	58944692	8.84G	57063118	8.56G	0.01	98.14	95.06	50
HM_2_BB	61788354	9.27G	59424120	8.91G	0.01	98.15	95.11	50.45
HM_2_T	62532088	9.38G	61430320	9.21G	0.01	98.53	95.49	47.69
HM_3_LD	66740976	10.01G	65390200	9.81G	0.01	98.75	96.4	49.61
HM_3_GM	61554414	9.23G	59991426	9.0G	0.01	98.61	95.97	51.59
HM_3_QF	53540290	8.03G	52201644	7.83G	0.01	98.22	94.66	52.29
HM_3_IM	58712644	8.81G	57768876	8.67G	0.01	98.81	96.64	51.82
HM_3_BB	61866918	9.28G	60425532	9.06G	0.01	98.66	96.14	51.61
HM_3_T	65679458	9.85G	64175692	9.63G	0.01	98.29	94.83	52.21
HM_4_LD	55044194	8.26G	53878290	8.08G	0.01	98.4	95.2	51.96
HM_4_GM	58176134	8.73G	56900840	8.54G	0.01	98.47	95.45	52.48
HM_4_QF	53679662	8.05G	52366968	7.86G	0.01	98.43	95.27	52.47
HM_4_IM	58631424	8.79G	57411490	8.61G	0.01	98.3	94.86	52.5
HM_4_BB	55668764	8.35G	54509594	8.18G	0.01	98.6	95.94	52.33
HM_4_T	59261712	8.89G	57952370	8.69G	0.01	98.83	96.75	52.95
HM_5_LD	55342254	8.3G	53999454	8.1G	0.01	98.46	95.49	52.42
HM_5_GM	67391134	10.11G	66645384	10.0G	0.01	98.64	95.77	47.61
HM_5_QF	54954996	8.24G	54226216	8.13G	0.01	98.64	95.94	49.56
HM_5_IM	57836256	8.68G	56436156	8.47G	0.01	98.26	94.8	51.71
HM_5_BB	53551650	8.03G	52506114	7.88G	0.01	98.35	95.06	52.47
HM_5_T	55703586	8.36G	54381938	8.16G	0.01	98.43	95.47	52.72
HM_6_LD	55407718	8.31G	54237874	8.14G	0.01	98.51	95.61	52.06
HM_6_GM	53337918	8G	52141116	7.82G	0.01	98.42	95.24	50.86
HM_6_QF	52989340	7.95G	52043364	7.81G	0.01	98.4	95.12	50.14
HM_6_IM	55194654	8.28G	53832444	8.07G	0.01	98.63	96.06	52.35
HM_6_BB	55382420	8.31G	53950754	8.09G	0.01	98.61	95.98	51.98
HM_6_T	55156310	8.27G	53761290	8.06G	0.01	98.6	95.96	52.7
