# Supplementary material for: Comparative transcriptome analysis reveals the patterns of gene expression in different venison cuts of sika deer (Cervus nippon)
Source: Anim Biosci. 2025 May 12;38(11):2324–35. doi: 10.5713/ab.25.0044 (PMC12580950; doi:10.5713/ab.25.0044)
Supplement: Supplementary file 2 [file ab-25-0044-supplementary-2.pdf]

Supplement 2. Statistics of sample mapping rate

Sample	total_reads	total_map	unique_map	multi_map	read1_map	read2_map	positive_map	negative_map	splice_map	unsplice_map	proper_map
HG_1_LD	60011646	56386929(93.96%)	51331368(85.54%)	5055561(8.42%)	25725719(42.87%)	25605649(42.67%)	25714421(42.85%)	25616947(42.69%)	25218795(42.02%)	26112573(43.51%)	47925362(79.86%)
HG_1_GM	54233864	50898919(93.85%)	45955773(84.74%)	4943146(9.11%)	23065693(42.53%)	22890080(42.21%)	23041301(42.49%)	22914472(42.25%)	21628902(39.88%)	24326871(44.86%)	42779418(78.88%)
HG_1_QF	58230416	54779023(94.07%)	50705705(87.08%)	4073318(7.0%)	25419245(43.65%)	25286460(43.42%)	25394009(43.61%)	25311696(43.47%)	23381245(40.15%)	27324460(46.92%)	47506748(81.58%)
HG_1_IM	54917540	52057933(94.79%)	48505312(88.32%)	3552621(6.47%)	24310774(44.27%)	24194538(44.06%)	24276296(44.2%)	24229016(44.12%)	21216170(38.63%)	27289142(49.69%)	45750370(83.31%)
HG_1_BB	55511236	52669587(94.88%)	46689567(84.11%)	5980020(10.77%)	23388678(42.13%)	23300889(41.98%)	23430090(42.21%)	23259477(41.9%)	17330953(31.22%)	29358614(52.89%)	43739642(78.79%)
HG_1_T	53150158	50425684(94.87%)	42995513(80.89%)	7430171(13.98%)	21526392(40.5%)	21469121(40.39%)	21616182(40.67%)	21379331(40.22%)	11422129(21.49%)	31573384(59.4%)	39819374(74.92%)
HG_2_LD	60346730	57479670(95.25%)	53300663(88.32%)	4179007(6.92%)	26694427(44.24%)	26606236(44.09%)	26677705(44.21%)	26622958(44.12%)	24151562(40.02%)	29149101(48.3%)	50772632(84.13%)
HG_2_GM	53654196	50224868(93.61%)	46799380(87.22%)	3425488(6.38%)	23456449(43.72%)	23342931(43.51%)	23423112(43.66%)	23376268(43.57%)	21302451(39.7%)	25496929(47.52%)	43681834(81.41%)
HG_2_QF	52590594	49321920(93.78%)	44622675(84.85%)	4699245(8.94%)	22399252(42.59%)	22223423(42.26%)	22352654(42.5%)	22270021(42.35%)	16449550(31.28%)	28173125(53.57%)	42121474(80.09%)
HG_2_IM	53278602	50046610(93.93%)	46495899(87.27%)	3550711(6.66%)	23306541(43.74%)	23189358(43.52%)	23268162(43.67%)	23227737(43.6%)	20502565(38.48%)	25993334(48.79%)	43726880(82.07%)
HG_2_BB	56287764	52725802(93.67%)	48612804(86.36%)	4112998(7.31%)	24358977(43.28%)	24253827(43.09%)	24311828(43.19%)	24300976(43.17%)	22485101(39.95%)	26127703(46.42%)	46641012(82.86%)
HG_2_T	51758450	48363257(93.44%)	44925228(86.8%)	3438029(6.64%)	22545440(43.56%)	22379788(43.24%)	22458761(43.39%)	22466467(43.41%)	20411085(39.44%)	24514143(47.36%)	40503914(78.26%)
HG_3_LD	53806282	51053462(94.88%)	48305223(89.78%)	2748239(5.11%)	24274603(45.11%)	24030620(44.66%)	24151906(44.89%)	24153317(44.89%)	18920835(35.16%)	29384388(54.61%)	45195992(84.0%)
HG_3_GM	59406174	56025789(94.31%)	53276167(89.68%)	2749622(4.63%)	26732116(45.0%)	26544051(44.68%)	26630485(44.83%)	26645682(44.85%)	22050655(37.12%)	31225512(52.56%)	49765502(83.77%)
HG_3_QF	53031692	49927736(94.15%)	46747451(88.15%)	3180285(6.0%)	23459565(44.24%)	23287886(43.91%)	23372819(44.07%)	23374632(44.08%)	18220083(34.36%)	28527368(53.79%)	42964902(81.02%)
HG_3_IM	53043642	49554845(93.42%)	46973094(88.56%)	2581751(4.87%)	23561392(44.42%)	23411702(44.14%)	23492945(44.29%)	23480149(44.27%)	18167674(34.25%)	28805420(54.31%)	43106458(81.27%)
HG_3_BB	53510328	50537891(94.45%)	48274102(90.21%)	2263789(4.23%)	24227185(45.28%)	24046917(44.94%)	24138091(45.11%)	24136011(45.11%)	19145641(35.78%)	29128461(54.44%)	44906186(83.92%)
HG_3_T	53030914	50480637(95.19%)	48365814(91.2%)	2114823(3.99%)	24266106(45.76%)	24099708(45.44%)	24177480(45.59%)	24188334(45.61%)	20073692(37.85%)	28292122(53.35%)	45854160(86.47%)
HG_4_LD	54281960	50624734(93.26%)	45970926(84.69%)	4653808(8.57%)	23084141(42.53%)	22886785(42.16%)	23005270(42.38%)	22965656(42.31%)	19643848(36.19%)	26327078(48.5%)	40966700(75.47%)
HG_4_GM	53061102	50433501(95.05%)	45874950(86.46%)	4558551(8.59%)	22988548(43.32%)	22886402(43.13%)	22997745(43.34%)	22877205(43.11%)	21138672(39.84%)	24736278(46.62%)	43058428(81.15%)
HG_4_QF	53236098	50116084(94.14%)	45364328(85.21%)	4751756(8.93%)	22784252(42.8%)	22580076(42.41%)	22744376(42.72%)	22619952(42.49%)	21587725(40.55%)	23776603(44.66%)	42182790(79.24%)
HG_4_IM	53222206	50450998(94.79%)	46344329(87.08%)	4106669(7.72%)	23232574(43.65%)	23111755(43.43%)	23203563(43.6%)	23140766(43.48%)	20934425(39.33%)	25409904(47.74%)	43392104(81.53%)
HG_4_BB	54386622	51143739(94.04%)	46874083(86.19%)	4269656(7.85%)	23543981(43.29%)	23330102(42.9%)	23465228(43.15%)	23408855(43.04%)	23588549(43.37%)	23285534(42.81%)	43888274(80.7%)
HG_4_T	53567042	50805413(94.84%)	46560747(86.92%)	4244666(7.92%)	23389180(43.66%)	23171567(43.26%)	23319184(43.53%)	23241563(43.39%)	20772635(38.78%)	25788112(48.14%)	43545562(81.29%)

HG_5_LD	54784558	51002368(93.1%)	47941998(87.51%)	3060370(5.59%)	24046519(43.89%)	23895479(43.62%)	23969589(43.75%)	23972409(43.76%)	20989939(38.31%)	26952059(49.2%)	43316518(79.07%)
HG_5_GM	57613508	54737386(95.01%)	51253877(88.96%)	3483509(6.05%)	25682711(44.58%)	25571166(44.38%)	25642672(44.51%)	25611205(44.45%)	23711508(41.16%)	27542369(47.81%)	48463306(84.12%)
HG_5_QF	66448088	61602320(92.71%)	56900447(85.63%)	4701873(7.08%)	28527338(42.93%)	28373109(42.7%)	28451117(42.82%)	28449330(42.81%)	23896099(35.96%)	33004348(49.67%)	50458156(75.94%)
HG_5_IM	53681646	49849135(92.86%)	46788038(87.16%)	3061097(5.7%)	23462586(43.71%)	23325452(43.45%)	23399942(43.59%)	23388096(43.57%)	20796750(38.74%)	25991288(48.42%)	41480824(77.27%)
HG_5_BB	52733568	50236830(95.27%)	47188399(89.48%)	3048431(5.78%)	23672149(44.89%)	23516250(44.59%)	23601330(44.76%)	23587069(44.73%)	22442288(42.56%)	24746111(46.93%)	44153830(83.73%)
HG_5_T	62332538	58997506(94.65%)	56336358(90.38%)	2661148(4.27%)	28275931(45.36%)	28060427(45.02%)	28160266(45.18%)	28176092(45.2%)	24229341(38.87%)	32107017(51.51%)	52364768(84.01%)
HG_6_LD	52750464	48742973(92.4%)	44997140(85.3%)	3745833(7.1%)	22551620(42.75%)	22445520(42.55%)	22525513(42.7%)	22471627(42.6%)	22289082(42.25%)	22708058(43.05%)	40705914(77.17%)
HG_6_GM	55282792	51987570(94.04%)	48253820(87.29%)	3733750(6.75%)	24289440(43.94%)	23964380(43.35%)	24132975(43.65%)	24120845(43.63%)	24013220(43.44%)	24240600(43.85%)	44953094(81.31%)
HG_6_QF	53872502	51122648(94.9%)	47538302(88.24%)	3584346(6.65%)	23845333(44.26%)	23692969(43.98%)	23800109(44.18%)	23738193(44.06%)	23718048(44.03%)	23820254(44.22%)	44095542(81.85%)
HG_6_IM	61242100	58348364(95.27%)	55076545(89.93%)	3271819(5.34%)	27612159(45.09%)	27464386(44.85%)	27545029(44.98%)	27531516(44.96%)	26947282(44.0%)	28129263(45.93%)	52320296(85.43%)
HG_6_BB	61288756	57034025(93.06%)	52592042(85.81%)	4441983(7.25%)	26392071(43.06%)	26199971(42.75%)	26329160(42.96%)	26262882(42.85%)	25426860(41.49%)	27165182(44.32%)	46808472(76.37%)
HG_6_T	56637520	53811730(95.01%)	51123062(90.26%)	2688668(4.75%)	25612771(45.22%)	25510291(45.04%)	25560860(45.13%)	25562202(45.13%)	23937231(42.26%)	27185831(48.0%)	48606820(85.82%)
HM_1_LD	55018806	52110188(94.71%)	49914372(90.72%)	2195816(3.99%)	25090921(45.6%)	24823451(45.12%)	24957902(45.36%)	24956470(45.36%)	20938097(38.06%)	28976275(52.67%)	46844666(85.14%)
HM_1_GM	66455434	62415656(93.92%)	58887388(88.61%)	3528268(5.31%)	29527047(44.43%)	29360341(44.18%)	29435157(44.29%)	29452231(44.32%)	23323877(35.1%)	35563511(53.51%)	54562036(82.1%)
HM_1_QF	54026308	50901499(94.22%)	48355597(89.5%)	2545902(4.71%)	24285656(44.95%)	24069941(44.55%)	24175734(44.75%)	24179863(44.76%)	19865649(36.77%)	28489948(52.73%)	44773164(82.87%)
HM_1_IM	64869216	61737877(95.17%)	58923185(90.83%)	2814692(4.34%)	29566615(45.58%)	29356570(45.26%)	29450668(45.4%)	29472517(45.43%)	23308767(35.93%)	35614418(54.9%)	55379226(85.37%)
HM_1_BB	61398572	58050622(94.55%)	55202015(89.91%)	2848607(4.64%)	27692003(45.1%)	27510012(44.81%)	27608568(44.97%)	27593447(44.94%)	24001668(39.09%)	31200347(50.82%)	51879736(84.5%)
HM_1_T	54147172	51151163(94.47%)	48855768(90.23%)	2295395(4.24%)	24508058(45.26%)	24347710(44.97%)	24422648(45.1%)	24433120(45.12%)	20926538(38.65%)	27929230(51.58%)	45410136(83.86%)
HM_2_LD	57050040	53593448(93.94%)	49359987(86.52%)	4233461(7.42%)	24752428(43.39%)	24607559(43.13%)	24726641(43.34%)	24633346(43.18%)	23118352(40.52%)	26241635(46.0%)	45510262(79.77%)
HM_2_GM	53491810	50840559(95.04%)	47853124(89.46%)	2987435(5.58%)	23992750(44.85%)	23860374(44.61%)	23921808(44.72%)	23931316(44.74%)	21566447(40.32%)	26286677(49.14%)	45247828(84.59%)
HM_2_QF	60524858	58227878(96.2%)	54395344(89.87%)	3832534(6.33%)	27254626(45.03%)	27140718(44.84%)	27236677(45.0%)	27158667(44.87%)	26201404(43.29%)	28193940(46.58%)	52235310(86.3%)
HM_2_IM	57063118	54520640(95.54%)	50111158(87.82%)	4409482(7.73%)	25348230(44.42%)	24762928(43.4%)	25129666(44.04%)	24981492(43.78%)	21597317(37.85%)	28513841(49.97%)	47810626(83.79%)
HM_2_BB	59424120	56803280(95.59%)	52399839(88.18%)	4403441(7.41%)	26496210(44.59%)	25903629(43.59%)	26256177(44.18%)	26143662(44.0%)	23258850(39.14%)	29140989(49.04%)	50017940(84.17%)
HM_2_T	61430320	58615564(95.42%)	50315029(81.91%)	8300535(13.51%)	25197279(41.02%)	25117750(40.89%)	25263253(41.13%)	25051776(40.78%)	14283831(23.25%)	36031198(58.65%)	47161144(76.77%)
HM_3_LD	65390200	61135001(93.49%)	56245037(86.01%)	4889964(7.48%)	28221982(43.16%)	28023055(42.86%)	28130629(43.02%)	28114408(42.99%)	24070279(36.81%)	32174758(49.2%)	53374894(81.63%)
HM_3_GM	59991426	56689473(94.5%)	53270817(88.8%)	3418656(5.7%)	26773126(44.63%)	26497691(44.17%)	26641940(44.41%)	26628877(44.39%)	22008375(36.69%)	31262442(52.11%)	49472190(82.47%)

HM_3_QF	52201644	49110282(94.08%)	46604580(89.28%)	2505702(4.8%)	23390865(44.81%)	23213715(44.47%)	23315279(44.66%)	23289301(44.61%)	19937853(38.19%)	26666727(51.08%)	43025382(82.42%)
HM_3_IM	57768876	54570322(94.46%)	51329785(88.85%)	3240537(5.61%)	25747330(44.57%)	25582455(44.28%)	25679864(44.45%)	25649921(44.4%)	21142627(36.6%)	30187158(52.26%)	48793262(84.46%)
HM_3_BB	60425532	57019992(94.36%)	53188461(88.02%)	3831531(6.34%)	26715269(44.21%)	26473192(43.81%)	26612337(44.04%)	26576124(43.98%)	22207064(36.75%)	30981397(51.27%)	49925086(82.62%)
HM_3_T	64175692	60996982(95.05%)	57278351(89.25%)	3718631(5.79%)	28729066(44.77%)	28549285(44.49%)	28647816(44.64%)	28630535(44.61%)	24469818(38.13%)	32808533(51.12%)	53474314(83.32%)
HM_4_LD	53878290	51231164(95.09%)	49053809(91.05%)	2177355(4.04%)	24599328(45.66%)	24454481(45.39%)	24525954(45.52%)	24527855(45.52%)	21880362(40.61%)	27173447(50.43%)	46544370(86.39%)
HM_4_GM	56900840	54003239(94.91%)	51880855(91.18%)	2122384(3.73%)	26044778(45.77%)	25836077(45.41%)	25929030(45.57%)	25951825(45.61%)	22942279(40.32%)	28938576(50.86%)	48524604(85.28%)
HM_4_QF	52366968	50046698(95.57%)	48165437(91.98%)	1881261(3.59%)	24148388(46.11%)	24017049(45.86%)	24076782(45.98%)	24088655(46.0%)	21646869(41.34%)	26518568(50.64%)	46006234(87.85%)
HM_4_IM	57411490	54931654(95.68%)	52982823(92.29%)	1948831(3.39%)	26588147(46.31%)	26394676(45.97%)	26481988(46.13%)	26500835(46.16%)	23119277(40.27%)	29863546(52.02%)	50096092(87.26%)
HM_4_BB	54509594	52116250(95.61%)	49614761(91.02%)	2501489(4.59%)	24903613(45.69%)	24711148(45.33%)	24807166(45.51%)	24807595(45.51%)	19456904(35.69%)	30157857(55.33%)	47748674(87.6%)
HM_4_T	57952370	54985019(94.88%)	52855859(91.21%)	2129160(3.67%)	26511565(45.75%)	26344294(45.46%)	26416870(45.58%)	26438989(45.62%)	23273703(40.16%)	29582156(51.05%)	50196088(86.62%)
HM_5_LD	53999454	51250998(94.91%)	48740213(90.26%)	2510785(4.65%)	24485860(45.34%)	24254353(44.92%)	24372554(45.13%)	24367659(45.13%)	20383457(37.75%)	28356756(52.51%)	45960414(85.11%)
HM_5_GM	66645384	61437167(92.19%)	58329584(87.52%)	3107583(4.66%)	29239673(43.87%)	29089911(43.65%)	29152484(43.74%)	29177100(43.78%)	20678223(31.03%)	37651361(56.5%)	53639476(80.48%)
HM_5_QF	54226216	50857876(93.79%)	47575929(87.74%)	3281947(6.05%)	23896834(44.07%)	23679095(43.67%)	23794846(43.88%)	23781083(43.86%)	19618623(36.18%)	27957306(51.56%)	43227784(79.72%)
HM_5_IM	56436156	52635297(93.27%)	50295236(89.12%)	2340061(4.15%)	25229618(44.7%)	25065618(44.41%)	25142055(44.55%)	25153181(44.57%)	18738720(33.2%)	31556516(55.92%)	47703758(84.53%)
HM_5_BB	52506114	49390486(94.07%)	47421699(90.32%)	1968787(3.75%)	23795341(45.32%)	23626358(45.0%)	23708045(45.15%)	23713654(45.16%)	17758361(33.82%)	29663338(56.5%)	45134314(85.96%)
HM_5_T	54381938	51216265(94.18%)	48405581(89.01%)	2810684(5.17%)	24336033(44.75%)	24069548(44.26%)	24196096(44.49%)	24209485(44.52%)	19967453(36.72%)	28438128(52.29%)	45245664(83.2%)
HM_6_LD	54237874	51334406(94.65%)	47980457(88.46%)	3353949(6.18%)	24099275(44.43%)	23881182(44.03%)	24021148(44.29%)	23959309(44.17%)	21757436(40.11%)	26223021(48.35%)	44479808(82.01%)
HM_6_GM	52141116	48384926(92.8%)	45182511(86.65%)	3202415(6.14%)	22649058(43.44%)	22533453(43.22%)	22617185(43.38%)	22565326(43.28%)	19983010(38.32%)	25199501(48.33%)	40773634(78.2%)
HM_6_QF	52043364	48523879(93.24%)	45388782(87.21%)	3135097(6.02%)	22765108(43.74%)	22623674(43.47%)	22719420(43.65%)	22669362(43.56%)	18975093(36.46%)	26413689(50.75%)	41062546(78.9%)
HM_6_IM	53832444	51103146(94.93%)	47660842(88.54%)	3442304(6.39%)	23912808(44.42%)	23748034(44.11%)	23836304(44.28%)	23824538(44.26%)	21584325(40.1%)	26076517(48.44%)	45681448(84.86%)
HM_6_BB	53950754	51122685(94.76%)	48111841(89.18%)	3010844(5.58%)	24142780(44.75%)	23969061(44.43%)	24071294(44.62%)	24040547(44.56%)	21423399(39.71%)	26688442(49.47%)	45589826(84.5%)
HM_6_T	53761290	50925649(94.73%)	47844743(88.99%)	3080906(5.73%)	24009925(44.66%)	23834818(44.33%)	23922580(44.5%)	23922163(44.5%)	21664151(40.3%)	26180592(48.7%)	45249752(84.17%)
