# Supplementary material for: Comparative transcriptome analysis reveals the patterns of gene expression in different venison cuts of sika deer (Cervus nippon)
Source: Anim Biosci. 2025 May 12;38(11):2324–35. doi: 10.5713/ab.25.0044 (PMC12580950; doi:10.5713/ab.25.0044)
Supplement: Supplementary file 3 [file ab-25-0044-supplementary-3.pdf]

Supplement 3. The KEGG enrichment results of DEGs between LD and T

KEGGID	Description	GeneRatio	BgRatio	pvalue
bta04340	Hedgehog signaling pathway	19/1208	52/7996	0.00011501
bta03050	Proteasome	17/1208	52/7996	0.001140726
bta04144	Endocytosis	58/1208	263/7996	0.001469435
bta00010	Glycolysis / Gluconeogenesis	21/1208	72/7996	0.001666955
bta00020	Citrate cycle (TCA cycle)	13/1208	40/7996	0.004490833
bta01200	Carbon metabolism	31/1208	129/7996	0.004840052
bta04261	Adrenergic signaling in cardiomyocytes	37/1208	165/7996	0.007496196
bta01522	Endocrine resistance	24/1208	97/7996	0.008462472
bta05163	Human cytomegalovirus infection	47/1208	223/7996	0.009557754
bta04915	Estrogen signaling pathway	30/1208	130/7996	0.010050786
bta04022	cGMP-PKG signaling pathway	39/1208	182/7996	0.013197267
bta04928	Parathyroid hormone synthesis, secretion and action	26/1208	112/7996	0.014673653
bta05170	Human immunodeficiency virus 1 infection	44/1208	212/7996	0.015477685
bta00785	Lipoic acid metabolism	8/1208	23/7996	0.015704878
bta04925	Aldosterone synthesis and secretion	24/1208	102/7996	0.015805621
bta04921	Oxytocin signaling pathway	36/1208	168/7996	0.016781595
bta00030	Pentose phosphate pathway	9/1208	28/7996	0.018321289
bta04750	Inflammatory mediator regulation of TRP channels	25/1208	109/7996	0.019082507
bta04137	Mitophagy - animal	26/1208	116/7996	0.022597342
bta00640	Propanoate metabolism	11/1208	39/7996	0.025903959
bta04550	Signaling pathways regulating pluripotency of stem cells	29/1208	134/7996	0.026200074
bta04922	Glucagon signaling pathway	25/1208	112/7996	0.026226435
bta04218	Cellular senescence	36/1208	174/7996	0.02783334
bta04310	Wnt signaling pathway	36/1208	174/7996	0.02783334
bta04390	Hippo signaling pathway	36/1208	174/7996	0.02783334
bta04670	Leukocyte transendothelial migration	25/1208	113/7996	0.029016003
bta04140	Autophagy - animal	38/1208	186/7996	0.02919489
bta05418	Fluid shear stress and atherosclerosis	30/1208	141/7996	0.029821061
bta00250	Alanine, aspartate and glutamate metabolism	10/1208	36/7996	0.036352986
bta01524	Platinum drug resistance	17/1208	72/7996	0.037044104
bta05017	Spinocerebellar ataxia	32/1208	155/7996	0.037489778
bta04919	Thyroid hormone signaling pathway	28/1208	133/7996	0.039494857
bta04935	Growth hormone synthesis, secretion and action	26/1208	122/7996	0.040273342
bta05012	Parkinson disease	55/1208	291/7996	0.042361453
bta04929	GnRH secretion	16/1208	68/7996	0.043456709
bta04120	Ubiquitin mediated proteolysis	33/1208	163/7996	0.044785137
bta04020	Calcium signaling pathway	53/1208	281/7996	0.047150766
bta05020	Prion disease	53/1208	281/7996	0.047150766
bta04350	TGF-beta signaling pathway	24/1208	113/7996	0.049058345
