# Supplementary material for: Comparative transcriptome analysis reveals the patterns of gene expression in different venison cuts of sika deer (Cervus nippon)
Source: Anim Biosci. 2025 May 12;38(11):2324–35. doi: 10.5713/ab.25.0044 (PMC12580950; doi:10.5713/ab.25.0044)
Supplement: Supplementary file 4 [file ab-25-0044-supplementary-4.pdf]

Supplement 4. The GO enrichment results of DEGs between LD and T

GOID	Description	GeneRatio	BgRatio	pvalue
GO:0072330	monocarboxylic acid biosynthetic process	10/813	15/5204	1.17E-05
GO:0019359	nicotinamide nucleotide biosynthetic process	10/813	16/5204	2.68E-05
GO:0016053	organic acid biosynthetic process	11/813	19/5204	2.86E-05
GO:0046394	carboxylic acid biosynthetic process	11/813	19/5204	2.86E-05
GO:0006090	pyruvate metabolic process	8/813	11/5204	3.65E-05
GO:0006096	glycolytic process	8/813	11/5204	3.65E-05
GO:0006165	nucleoside diphosphate phosphorylation	8/813	11/5204	3.65E-05
GO:0006757	ATP generation from ADP	8/813	11/5204	3.65E-05
GO:0009132	nucleoside diphosphate metabolic process	8/813	11/5204	3.65E-05
GO:0009135	purine nucleoside diphosphate metabolic process	8/813	11/5204	3.65E-05
GO:0009179	purine ribonucleoside diphosphate metabolic process	8/813	11/5204	3.65E-05
GO:0009185	ribonucleoside diphosphate metabolic process	8/813	11/5204	3.65E-05
GO:0042866	pyruvate biosynthetic process	8/813	11/5204	3.65E-05
GO:0046031	ADP metabolic process	8/813	11/5204	3.65E-05
GO:0046939	nucleotide phosphorylation	8/813	11/5204	3.65E-05
GO:0019363	pyridine nucleotide biosynthetic process	10/813	17/5204	5.59E-05
GO:0072525	pyridine-containing compound biosynthetic process	10/813	17/5204	5.59E-05
GO:0016052	carbohydrate catabolic process	8/813	12/5204	9.45E-05
GO:0019362	pyridine nucleotide metabolic process	10/813	19/5204	0.000196771
GO:0046496	nicotinamide nucleotide metabolic process	10/813	19/5204	0.000196771
GO:0072524	pyridine-containing compound metabolic process	10/813	19/5204	0.000196771
GO:0009152	purine ribonucleotide biosynthetic process	16/813	41/5204	0.000236615
GO:0009260	ribonucleotide biosynthetic process	16/813	41/5204	0.000236615
GO:0046390	ribose phosphate biosynthetic process	16/813	41/5204	0.000236615
GO:0032787	monocarboxylic acid metabolic process	11/813	23/5204	0.000279824
GO:0035556	intracellular signal transduction	60/813	252/5204	0.000331507
GO:0006022	aminoglycan metabolic process	7/813	11/5204	0.000410122
GO:0030203	glycosaminoglycan metabolic process	7/813	11/5204	0.000410122
GO:0009165	nucleotide biosynthetic process	22/813	69/5204	0.000529201
GO:1901293	nucleoside phosphate biosynthetic process	22/813	69/5204	0.000529201
GO:0006733	oxidoreduction coenzyme metabolic process	10/813	21/5204	0.000557262
GO:0019752	carboxylic acid metabolic process	27/813	92/5204	0.000575949
GO:0006164	purine nucleotide biosynthetic process	16/813	44/5204	0.000603196
GO:0006754	ATP biosynthetic process	11/813	25/5204	0.000682987
GO:0009142	nucleoside triphosphate biosynthetic process	11/813	25/5204	0.000682987
GO:0009145	purine nucleoside triphosphate biosynthetic process	11/813	25/5204	0.000682987
GO:0009201	ribonucleoside triphosphate biosynthetic process	11/813	25/5204	0.000682987
GO:0009206	purine ribonucleoside triphosphate biosynthetic process	11/813	25/5204	0.000682987
GO:0006082	organic acid metabolic process	27/813	93/5204	0.000693953
GO:0043436	oxoacid metabolic process	27/813	93/5204	0.000693953
GO:0072522	purine-containing compound biosynthetic process	16/813	45/5204	0.000802846
GO:0017144	drug metabolic process	18/813	54/5204	0.000931878

GO:0009166	nucleotide catabolic process	8/813	16/5204	0.001367432
GO:0090407	organophosphate biosynthetic process	28/813	102/5204	0.001454236
GO:0006091	generation of precursor metabolites and energy	11/813	27/5204	0.001480391
GO:0009124	nucleoside monophosphate biosynthetic process	11/813	27/5204	0.001480391
GO:0009127	purine nucleoside monophosphate biosynthetic process	11/813	27/5204	0.001480391
GO:0009156	ribonucleoside monophosphate biosynthetic process	11/813	27/5204	0.001480391
GO:0009168	purine ribonucleoside monophosphate biosynthetic process	11/813	27/5204	0.001480391
GO:0009108	coenzyme biosynthetic process	12/813	31/5204	0.001544038
GO:0044281	small molecule metabolic process	52/813	225/5204	0.001677314
GO:0009150	purine ribonucleotide metabolic process	18/813	57/5204	0.00187983
GO:0009259	ribonucleotide metabolic process	18/813	57/5204	0.00187983
GO:0019693	ribose phosphate metabolic process	18/813	59/5204	0.002884545
GO:0009966	regulation of signal transduction	33/813	132/5204	0.003146608
GO:0010646	regulation of cell communication	33/813	132/5204	0.003146608
GO:1901292	nucleoside phosphate catabolic process	8/813	18/5204	0.003476011
GO:0009117	nucleotide metabolic process	25/813	93/5204	0.003482727
GO:0006163	purine nucleotide metabolic process	18/813	60/5204	0.003534469
GO:0023051	regulation of signaling	33/813	133/5204	0.003577613
GO:0009056	catabolic process	33/813	134/5204	0.004057874
GO:0072521	purine-containing compound metabolic process	18/813	61/5204	0.004301184
GO:0048583	regulation of response to stimulus	34/813	140/5204	0.004520227
GO:0046034	ATP metabolic process	13/813	39/5204	0.004666074
GO:0006753	nucleoside phosphate metabolic process	25/813	95/5204	0.004718872
GO:1902531	regulation of intracellular signal transduction	25/813	95/5204	0.004718872
GO:0044248	cellular catabolic process	29/813	115/5204	0.004778639
GO:0006732	coenzyme metabolic process	12/813	35/5204	0.005009479
GO:0034404	nucleobase-containing small molecule biosynthetic process	8/813	19/5204	0.005194348
GO:1901575	organic substance catabolic process	31/813	126/5204	0.005340592
GO:0006508	proteolysis	74/813	360/5204	0.005825742
GO:0009144	purine nucleoside triphosphate metabolic process	13/813	40/5204	0.005947682
GO:0009199	ribonucleoside triphosphate metabolic process	13/813	40/5204	0.005947682
GO:0009205	purine ribonucleoside triphosphate metabolic process	13/813	40/5204	0.005947682
GO:0009057	macromolecule catabolic process	20/813	73/5204	0.006786924
GO:0030163	protein catabolic process	17/813	59/5204	0.007085374
GO:0009123	nucleoside monophosphate metabolic process	13/813	41/5204	0.007494916
GO:0009126	purine nucleoside monophosphate metabolic process	13/813	41/5204	0.007494916
GO:0009161	ribonucleoside monophosphate metabolic process	13/813	41/5204	0.007494916
GO:0009167	purine ribonucleoside monophosphate metabolic process	13/813	41/5204	0.007494916
GO:0051186	cofactor metabolic process	16/813	55/5204	0.008026405
GO:1901565	organonitrogen compound catabolic process	21/813	79/5204	0.008137228
GO:0051188	cofactor biosynthetic process	12/813	37/5204	0.008249084
GO:0051056	regulation of small GTPase mediated signal transduction	23/813	89/5204	0.008302033
GO:0006511	ubiquitin-dependent protein catabolic process	13/813	42/5204	0.009343438
GO:0009141	nucleoside triphosphate metabolic process	13/813	42/5204	0.009343438

GO:0019941	modification-dependent protein catabolic process	13/813	42/5204	0.009343438
GO:0043632	modification-dependent macromolecule catabolic process	13/813	42/5204	0.009343438
GO:0044283	small molecule biosynthetic process	13/813	42/5204	0.009343438
GO:0032879	regulation of localization	7/813	17/5204	0.010232578
GO:0007264	small GTPase mediated signal transduction	28/813	116/5204	0.010310098
GO:0009190	cyclic nucleotide biosynthetic process	7/813	18/5204	0.014532211
GO:0005975	carbohydrate metabolic process	28/813	119/5204	0.01458292
GO:0055086	nucleobase-containing small molecule metabolic process	25/813	105/5204	0.017573259
GO:0044257	cellular protein catabolic process	15/813	55/5204	0.018532469
GO:0051603	proteolysis involved in cellular protein catabolic process	15/813	55/5204	0.018532469
GO:0007266	Rho protein signal transduction	17/813	65/5204	0.019161983
GO:0035023	regulation of Rho protein signal transduction	17/813	65/5204	0.019161983
GO:0009187	cyclic nucleotide metabolic process	7/813	19/5204	0.019979298
GO:0048193	Golgi vesicle transport	7/813	19/5204	0.019979298
GO:0044265	cellular macromolecule catabolic process	17/813	66/5204	0.02218449
GO:0034655	nucleobase-containing compound catabolic process	9/813	28/5204	0.022369177
GO:0046434	organophosphate catabolic process	8/813	24/5204	0.024643948
GO:0007265	Ras protein signal transduction	19/813	78/5204	0.028499442
GO:0046578	regulation of Ras protein signal transduction	19/813	78/5204	0.028499442
GO:0009893	positive regulation of metabolic process	8/813	25/5204	0.031431002
GO:0010604	positive regulation of macromolecule metabolic process	8/813	25/5204	0.031431002
GO:0031325	positive regulation of cellular metabolic process	8/813	25/5204	0.031431002
GO:0051173	positive regulation of nitrogen compound metabolic process	8/813	25/5204	0.031431002
GO:1901135	carbohydrate derivative metabolic process	33/813	155/5204	0.035113653
GO:0019439	aromatic compound catabolic process	10/813	36/5204	0.044288131
GO:1901361	organic cyclic compound catabolic process	10/813	36/5204	0.044288131
GO:0051641	cellular localization	28/813	131/5204	0.047320915
GO:0032268	regulation of cellular protein metabolic process	6/813	18/5204	0.049536663
GO:0098791	Golgi subcompartment	6/452	16/3235	0.016694417
GO:0000139	Golgi membrane	5/452	15/3235	0.047089673
GO:0004222	metalloendopeptidase activity	21/1349	71/8344	0.003236315
GO:0008081	phosphoric diester hydrolase activity	15/1349	46/8344	0.004423366
GO:0098772	molecular function regulator	79/1349	373/8344	0.005497397
GO:0000287	magnesium ion binding	10/1349	27/8344	0.007075912
GO:0008237	metallopeptidase activity	26/1349	101/8344	0.00886828
GO:0016763	transferase activity, transferring pentosyl groups	10/1349	28/8344	0.009443818
GO:0019208	phosphatase regulator activity	5/1349	10/8344	0.013542831
GO:0015085	calcium ion transmembrane transporter activity	6/1349	14/8344	0.016437139
GO:0016849	phosphorus-oxygen lyase activity	7/1349	18/8344	0.017497421
GO:0030695	GTPase regulator activity	14/1349	49/8344	0.020087884
GO:0005509	calcium ion binding	62/1349	300/8344	0.021308253
GO:0005262	calcium channel activity	5/1349	11/8344	0.021570648
GO:0019899	enzyme binding	33/1349	145/8344	0.023076878
GO:0017048	Rho GTPase binding	19/1349	74/8344	0.023708303

GO:0060589	nucleoside-triphosphatase regulator activity	15/1349	55/8344	0.024969426
GO:0005088	Ras guanyl-nucleotide exchange factor activity	17/1349	65/8344	0.026412466
GO:0005089	Rho guanyl-nucleotide exchange factor activity	17/1349	65/8344	0.026412466
GO:0003950	NAD+ ADP-ribosyltransferase activity	6/1349	16/8344	0.032886809
GO:0017016	Ras GTPase binding	21/1349	87/8344	0.034571031
GO:0031267	small GTPase binding	21/1349	87/8344	0.034571031
GO:0030234	enzyme regulator activity	31/1349	139/8344	0.034962512
GO:0005085	guanyl-nucleotide exchange factor activity	24/1349	104/8344	0.041055691
GO:0070011	peptidase activity, acting on L-amino acid peptides	69/1349	351/8344	0.043345374
GO:0004114	3',5'-cyclic-nucleotide phosphodiesterase activity	6/1349	17/8344	0.044067982
GO:0051020	GTPase binding	28/1349	126/8344	0.045203085
GO:0004435	phosphatidylinositol phospholipase C activity	5/1349	13/8344	0.045461832
GO:0004629	phospholipase C activity	5/1349	13/8344	0.045461832
