# Supplementary material for: Comparative transcriptome analysis reveals the patterns of gene expression in different venison cuts of sika deer (Cervus nippon)
Source: Anim Biosci. 2025 May 12;38(11):2324–35. doi: 10.5713/ab.25.0044 (PMC12580950; doi:10.5713/ab.25.0044)
Supplement: Supplementary file 5 [file ab-25-0044-supplementary-5.pdf]

Supplement 5. The KEGG enrichment results of DEGs between LD and QF

KEGGID	Description	GeneRatio	BgRatio	pvalue
bta04814	Motor proteins	9/89	211/7974	0.000544588
bta04145	Phagosome	6/89	165/7974	0.010130537
bta04977	Vitamin digestion and absorption	2/89	19/7974	0.0186217
bta04962	Vasopressin-regulated water reabsorption	3/89	52/7974	0.02004245
bta04640	Hematopoietic cell lineage	4/89	94/7974	0.020654132
bta00603	Glycosphingolipid biosynthesis - globo and isoglobo series	2/89	22/7974	0.02461871
bta00052	Galactose metabolism	2/89	24/7974	0.028995419
bta04914	Progesterone-mediated oocyte maturation	4/89	105/7974	0.029542399
bta05145	Toxoplasmosis	4/89	109/7974	0.033258145
bta05132	Salmonella infection	7/89	282/7974	0.037649625
bta05134	Legionellosis	3/89	67/7974	0.038586448
bta05310	Asthma	2/89	28/7974	0.038590366
bta00601	Glycosphingolipid biosynthesis - lacto and neolacto series	2/89	30/7974	0.043780048
bta00010	Glycolysis / Gluconeogenesis	3/89	73/7974	0.047789276
bta05133	Pertussis	3/89	73/7974	0.047789276
