# Supplementary material for: Comparative transcriptome analysis reveals the patterns of gene expression in different venison cuts of sika deer (Cervus nippon)
Source: Anim Biosci. 2025 May 12;38(11):2324–35. doi: 10.5713/ab.25.0044 (PMC12580950; doi:10.5713/ab.25.0044)
Supplement: Supplementary file 6 [file ab-25-0044-supplementary-6.pdf]

Supplement 6. The GO enrichment results of DEGs between LD and QF

GOID	Description	GeneRatio	BgRatio	pvalue
GO:0006955	immune response	5/60	92/5177	0.004017528
GO:0002376	immune system process	5/60	96/5177	0.004820684
GO:0072330	monocarboxylic acid biosynthetic process	2/60	15/5177	0.012588737
GO:0009190	cyclic nucleotide biosynthetic process	2/60	18/5177	0.017940064
GO:0009187	cyclic nucleotide metabolic process	2/60	19/5177	0.019902791
GO:0016053	organic acid biosynthetic process	2/60	19/5177	0.019902791
GO:0046394	carboxylic acid biosynthetic process	2/60	19/5177	0.019902791
GO:0006813	potassium ion transport	2/60	21/5177	0.024083294
GO:0007018	microtubule-based movement	3/60	57/5177	0.027819695
GO:0032787	monocarboxylic acid metabolic process	2/60	23/5177	0.028589512
GO:0055086	nucleobase-containing small molecule metabolic process	4/60	105/5177	0.032728507
GO:0006928	movement of cell or subcellular component	3/60	66/5177	0.040454775
GO:0009165	nucleotide biosynthetic process	3/60	68/5177	0.043597203
GO:1901293	nucleoside phosphate biosynthetic process	3/60	68/5177	0.043597203
GO:0005975	carbohydrate metabolic process	4/60	117/5177	0.045858767
GO:0055085	transmembrane transport	9/60	415/5177	0.047749975
GO:0005044	scavenger receptor activity	3/107	25/8316	0.003876745
GO:0038024	cargo receptor activity	3/107	25/8316	0.003876745
GO:0003774	motor activity	5/107	90/8316	0.005937307
GO:0005164	tumor necrosis factor receptor binding	2/107	13/8316	0.011663086
GO:0032813	tumor necrosis factor receptor superfamily binding	2/107	13/8316	0.011663086
GO:0016849	phosphorus-oxygen lyase activity	2/107	18/8316	0.021942505
GO:0005267	potassium channel activity	2/107	19/8316	0.02432076
GO:0015079	potassium ion transmembrane transporter activity	2/107	19/8316	0.02432076
GO:0003777	microtubule motor activity	3/107	53/8316	0.030486701
GO:0030234	enzyme regulator activity	5/107	139/8316	0.033254826
