# Supplementary material for: Comparative transcriptome analysis reveals the patterns of gene expression in different venison cuts of sika deer (Cervus nippon)
Source: Anim Biosci. 2025 May 12;38(11):2324–35. doi: 10.5713/ab.25.0044 (PMC12580950; doi:10.5713/ab.25.0044)
Supplement: Supplementary file 7 [file ab-25-0044-supplementary-7.pdf]

Supplement 7. The KEGG enrichment results of DEGs between LD and IM

KEGGID	Description	GeneRatio	BgRatio	pvalue
bta04340	Hedgehog signaling pathway	17/788	52/8017	5.03E-06
bta04974	Protein digestion and absorption	21/788	97/8017	0.000384841
bta04310	Wnt signaling pathway	30/788	172/8017	0.00126816
bta04350	TGF-beta signaling pathway	22/788	113/8017	0.001292129
bta04152	AMPK signaling pathway	25/788	136/8017	0.0014776
bta04933	AGE-RAGE signaling pathway in diabetic complications	21/788	107/8017	0.001484336
bta04512	ECM-receptor interaction	18/788	90/8017	0.002533532
bta04610	Complement and coagulation cascades	16/788	77/8017	0.002877164
bta04928	Parathyroid hormone synthesis, secretion and action	21/788	113/8017	0.002988191
bta04550	Signaling pathways regulating pluripotency of stem cells	24/788	136/8017	0.003191869
bta04979	Cholesterol metabolism	11/788	45/8017	0.003443402
bta04923	Regulation of lipolysis in adipocytes	13/788	58/8017	0.003487235
bta04918	Thyroid hormone synthesis	16/788	79/8017	0.003769296
bta04151	PI3K-Akt signaling pathway	51/788	358/8017	0.003947537
bta05165	Human papillomavirus infection	47/788	332/8017	0.006235639
bta05217	Basal cell carcinoma	13/788	62/8017	0.006364248
bta04390	Hippo signaling pathway	28/788	175/8017	0.006402727
bta05144	Malaria	10/788	48/8017	0.016489626
bta04926	Relaxin signaling pathway	21/788	132/8017	0.017809317
bta05207	Chemical carcinogenesis - receptor activation	27/788	183/8017	0.020425104
bta04911	Insulin secretion	15/788	87/8017	0.021454396
bta04510	Focal adhesion	29/788	202/8017	0.023452046
bta05226	Gastric cancer	22/788	144/8017	0.023910999
bta04211	Longevity regulating pathway	16/788	96/8017	0.024096032
bta04934	Cushing syndrome	24/788	161/8017	0.024798084
bta03050	Proteasome	10/788	52/8017	0.027958205
bta05218	Melanoma	12/788	67/8017	0.028564831
bta01522	Endocrine resistance	16/788	98/8017	0.028709469
bta04392	Hippo signaling pathway - multiple species	8/788	38/8017	0.028733131
bta05225	Hepatocellular carcinoma	24/788	164/8017	0.030202838
bta04917	Prolactin signaling pathway	12/788	68/8017	0.031707174
bta05212	Pancreatic cancer	13/788	77/8017	0.036040797
bta04261	Adrenergic signaling in cardiomyocytes	24/788	167/8017	0.036458409
bta04927	Cortisol synthesis and secretion	12/788	70/8017	0.038721417
bta05213	Endometrial cancer	11/788	63/8017	0.041465681
bta04360	Axon guidance	27/788	195/8017	0.041866323
