# Supplementary material for: Comparative transcriptome analysis reveals the patterns of gene expression in different venison cuts of sika deer (Cervus nippon)
Source: Anim Biosci. 2025 May 12;38(11):2324–35. doi: 10.5713/ab.25.0044 (PMC12580950; doi:10.5713/ab.25.0044)
Supplement: Supplementary file 8 [file ab-25-0044-supplementary-8.pdf]

Supplement 8. The GO enrichment results of DEGs between LD and IM

GOID	Description	GeneRatio	BgRatio	pvalue
GO:0007167	enzyme linked receptor protein signaling pathway	7/515	16/5213	0.000455604
GO:0071495	cellular response to endogenous stimulus	6/515	16/5213	0.00304527
GO:0009719	response to endogenous stimulus	6/515	17/5213	0.004319469
GO:0010033	response to organic substance	7/515	23/5213	0.005287583
GO:0007155	cell adhesion	21/515	119/5213	0.005745354
GO:0022610	biological adhesion	21/515	119/5213	0.005745354
GO:0070887	cellular response to chemical stimulus	6/515	19/5213	0.007981406
GO:0071310	cellular response to organic substance	6/515	19/5213	0.007981406
GO:0042221	response to chemical	8/515	32/5213	0.010655559
GO:0007166	cell surface receptor signaling pathway	14/515	75/5213	0.01376285
GO:0006979	response to oxidative stress	5/515	16/5213	0.016029605
GO:0006508	proteolysis	48/515	359/5213	0.016351036
GO:0051641	cellular localization	21/515	131/5213	0.016882252
GO:0008104	protein localization	19/515	117/5213	0.019731319
GO:0006886	intracellular protein transport	16/515	94/5213	0.020594114
GO:0034613	cellular protein localization	17/515	102/5213	0.021029574
GO:0070727	cellular macromolecule localization	17/515	102/5213	0.021029574
GO:0015031	protein transport	18/515	110/5213	0.021301642
GO:0015833	peptide transport	18/515	110/5213	0.021301642
GO:0046907	intracellular transport	18/515	110/5213	0.021301642
GO:0051649	establishment of localization in cell	18/515	111/5213	0.023180712
GO:0042886	amide transport	18/515	112/5213	0.025183464
GO:0045184	establishment of protein localization	18/515	112/5213	0.025183464
GO:0071705	nitrogen compound transport	20/515	130/5213	0.029125632
GO:0005576	extracellular region	40/319	215/3253	3.09E-05
GO:0044421	extracellular region part	10/319	43/3253	0.007201026
GO:0031012	extracellular matrix	5/319	17/3253	0.020185458
GO:0099080	supramolecular complex	5/319	17/3253	0.020185458
GO:0099081	supramolecular polymer	5/319	17/3253	0.020185458
GO:0099512	supramolecular fiber	5/319	17/3253	0.020185458
GO:0005875	microtubule associated complex	5/319	20/3253	0.039651476
GO:0005794	Golgi apparatus	7/319	35/3253	0.049588211
GO:0012505	endomembrane system	17/319	114/3253	0.04993861
GO:0005509	calcium ion binding	54/861	304/8361	3.83E-05
GO:0004222	metalloendopeptidase activity	18/861	70/8361	0.000185052
GO:0008237	metallopeptidase activity	23/861	103/8361	0.000254239
GO:0005201	extracellular matrix structural constituent	8/861	19/8361	0.000328048
GO:0004175	endopeptidase activity	36/861	227/8361	0.005461215
GO:0004601	peroxidase activity	5/861	15/8361	0.014260579
GO:0005520	insulin-like growth factor binding	5/861	17/8361	0.024718192
GO:0008233	peptidase activity	49/861	361/8361	0.025656947
GO:0070011	peptidase activity, acting on L-amino acid peptides	47/861	350/8361	0.033548369

GO:0004435	phosphatidylinositol phospholipase C activity	4/861	13/8361	0.037435252
GO:0004629	phospholipase C activity	4/861	13/8361	0.037435252
GO:0008536	Ran GTPase binding	4/861	13/8361	0.037435252
GO:0051287	NAD binding	6/861	25/8361	0.037624413
GO:0008081	phosphoric diester hydrolase activity	9/861	46/8361	0.042250986
GO:0004181	metallocarboxypeptidase activity	5/861	20/8361	0.047884881
GO:0008235	metalloexopeptidase activity	5/861	20/8361	0.047884881
GO:0019838	growth factor binding	5/861	20/8361	0.047884881
GO:0004842	ubiquitin-protein transferase activity	8/861	40/8361	0.048103118
GO:0019787	ubiquitin-like protein transferase activity	8/861	40/8361	0.048103118
