# Supplementary material for: Comparative transcriptome analysis reveals the patterns of gene expression in different venison cuts of sika deer (Cervus nippon)
Source: Anim Biosci. 2025 May 12;38(11):2324–35. doi: 10.5713/ab.25.0044 (PMC12580950; doi:10.5713/ab.25.0044)
Supplement: Supplementary file 9 [file ab-25-0044-supplementary-9.pdf]

Supplement 9. The KEGG enrichment results of DEGs between LD and BB

KEGGID	Description	GeneRatio	BgRatio	pvalue
bta00970	Aminoacyl-tRNA biosynthesis	9/512	45/8000	0.001870057
bta04550	Signaling pathways regulating pluripotency of stem cells	18/512	135/8000	0.002303584
bta05132	Salmonella infection	31/512	284/8000	0.002330601
bta04145	Phagosome	20/512	166/8000	0.004502073
bta04151	PI3K-Akt signaling pathway	36/512	359/8000	0.004561003
bta04962	Vasopressin-regulated water reabsorption	9/512	52/8000	0.005214157
bta04360	Axon guidance	22/512	192/8000	0.005454957
bta03450	Non-homologous end-joining	4/512	13/8000	0.007450726
bta05223	Non-small cell lung cancer	12/512	87/8000	0.009045757
bta05216	Thyroid cancer	8/512	48/8000	0.010385878
bta05235	PD-L1 expression and PD-1 checkpoint pathway in cancer	12/512	91/8000	0.012775711
bta05418	Fluid shear stress and atherosclerosis	16/512	142/8000	0.018974967
bta00534	Glycosaminoglycan biosynthesis - heparan sulfate / heparin	5/512	25/8000	0.019233866
bta01522	Endocrine resistance	12/512	97/8000	0.020444337
bta04512	ECM-receptor interaction	11/512	89/8000	0.025979865
bta04310	Wnt signaling pathway	18/512	173/8000	0.027794331
bta00532	Glycosaminoglycan biosynthesis - chondroitin sulfate / dermatan sulfate	4/512	20/8000	0.035404667
bta04611	Platelet activation	14/512	129/8000	0.035854576
bta04330	Notch signaling pathway	9/512	72/8000	0.039201691
bta04810	Regulation of actin cytoskeleton	22/512	233/8000	0.042417478
bta04064	NF-kappa B signaling pathway	12/512	110/8000	0.048006517
bta04964	Proximal tubule bicarbonate reclamation	4/512	22/8000	0.048357709
bta05320	Autoimmune thyroid disease	6/512	42/8000	0.049249193
