# Supplementary material for: Comparative transcriptome analysis reveals the patterns of gene expression in different venison cuts of sika deer (Cervus nippon)
Source: Anim Biosci. 2025 May 12;38(11):2324–35. doi: 10.5713/ab.25.0044 (PMC12580950; doi:10.5713/ab.25.0044)
Supplement: Supplementary file 10 [file ab-25-0044-supplementary-10.pdf]

Supplement 10. The GO enrichment results of DEGs between LD and BB

GOID	Description	GeneRatio	BgRatio	pvalue
GO:0006418	tRNA aminoacylation for protein translation	9/349	33/5213	0.000222763
GO:0043038	amino acid activation	9/349	36/5213	0.000454527
GO:0043039	tRNA aminoacylation	9/349	36/5213	0.000454527
GO:0006520	cellular amino acid metabolic process	11/349	56/5213	0.001000838
GO:0007266	Rho protein signal transduction	12/349	65/5213	0.001063768
GO:0035023	regulation of Rho protein signal transduction	12/349	65/5213	0.001063768
GO:0016192	vesicle-mediated transport	16/349	107/5213	0.001821749
GO:0008104	protein localization	17/349	117/5213	0.001826038
GO:0015031	protein transport	16/349	110/5213	0.002439842
GO:0015833	peptide transport	16/349	110/5213	0.002439842
GO:0051641	cellular localization	18/349	131/5213	0.002565638
GO:0019752	carboxylic acid metabolic process	14/349	91/5213	0.002641358
GO:0006082	organic acid metabolic process	14/349	92/5213	0.002931205
GO:0043436	oxoacid metabolic process	14/349	92/5213	0.002931205
GO:0042886	amide transport	16/349	112/5213	0.002941928
GO:0045184	establishment of protein localization	16/349	112/5213	0.002941928
GO:0034613	cellular protein localization	15/349	102/5213	0.002962377
GO:0070727	cellular macromolecule localization	15/349	102/5213	0.002962377
GO:0006886	intracellular protein transport	14/349	94/5213	0.003589396
GO:0007264	small GTPase mediated signal transduction	16/349	115/5213	0.003853239
GO:0007265	Ras protein signal transduction	12/349	78/5213	0.005225304
GO:0046578	regulation of Ras protein signal transduction	12/349	78/5213	0.005225304
GO:0051056	regulation of small GTPase mediated signal transduction	13/349	89/5213	0.005843148
GO:0046907	intracellular transport	15/349	110/5213	0.006126479
GO:0033036	macromolecule localization	19/349	153/5213	0.006178771
GO:0009966	regulation of signal transduction	17/349	132/5213	0.006530691
GO:0010646	regulation of cell communication	17/349	132/5213	0.006530691
GO:0051649	establishment of localization in cell	15/349	111/5213	0.006664814
GO:0023051	regulation of signaling	17/349	133/5213	0.007042634
GO:1902531	regulation of intracellular signal transduction	13/349	95/5213	0.010091261
GO:0006399	tRNA metabolic process	10/349	65/5213	0.010408951
GO:0048583	regulation of response to stimulus	17/349	140/5213	0.011601613
GO:0071705	nitrogen compound transport	16/349	130/5213	0.012498009
GO:0071702	organic substance transport	18/349	162/5213	0.022337589
GO:0035556	intracellular signal transduction	25/349	251/5213	0.028032559
GO:0017038	protein import	3/349	11/5213	0.032800445
GO:0006887	exocytosis	4/349	19/5213	0.0343215
GO:0032940	secretion by cell	4/349	19/5213	0.0343215
GO:0007018	microtubule-based movement	8/349	57/5213	0.034520336
GO:0034622	cellular protein-containing complex assembly	10/349	79/5213	0.036691887
GO:0034660	ncRNA metabolic process	10/349	82/5213	0.045709739
GO:0008380	RNA splicing	4/349	21/5213	0.047726574

GO:0007017	microtubule-based process	10/349	83/5213	0.049021997
GO:0015630	microtubule cytoskeleton	8/182	35/3244	0.000535114
GO:0005875	microtubule associated complex	6/182	20/3244	0.000574945
GO:0030286	dynein complex	4/182	16/3244	0.010244075
GO:0099023	tethering complex	4/182	16/3244	0.010244075
GO:0030117	membrane coat	5/182	27/3244	0.015477565
GO:0048475	coated membrane	5/182	27/3244	0.015477565
GO:0044431	Golgi apparatus part	5/182	30/3244	0.023855769
GO:0044430	cytoskeletal part	10/182	89/3244	0.025682095
GO:0005856	cytoskeleton	11/182	106/3244	0.033405126
GO:0005794	Golgi apparatus	5/182	35/3244	0.043410319
GO:0000139	Golgi membrane	3/182	15/3244	0.047985687
GO:0017016	Ras GTPase binding	17/569	87/8341	6.54E-05
GO:0031267	small GTPase binding	17/569	87/8341	6.54E-05
GO:0016874	ligase activity	13/569	67/8341	0.000500906
GO:0004812	aminoacyl-tRNA ligase activity	9/569	37/8341	0.000663366
GO:0016875	ligase activity, forming carbon-oxygen bonds	9/569	37/8341	0.000663366
GO:0019899	enzyme binding	21/569	144/8341	0.000720832
GO:0008536	Ran GTPase binding	5/569	13/8341	0.00117892
GO:0005088	Ras guanyl-nucleotide exchange factor activity	12/569	65/8341	0.001292713
GO:0005089	Rho guanyl-nucleotide exchange factor activity	12/569	65/8341	0.001292713
GO:0051020	GTPase binding	18/569	125/8341	0.001953378
GO:0140101	catalytic activity, acting on a tRNA	10/569	54/8341	0.003138304
GO:0017048	Rho GTPase binding	12/569	74/8341	0.004022512
GO:0005044	scavenger receptor activity	6/569	24/8341	0.004577276
GO:0038024	cargo receptor activity	6/569	24/8341	0.004577276
GO:0004842	ubiquitin-protein transferase activity	8/569	40/8341	0.004900184
GO:0019787	ubiquitin-like protein transferase activity	8/569	40/8341	0.004900184
GO:0016763	transferase activity, transferring pentosyl groups	6/569	28/8341	0.010157055
GO:0030695	GTPase regulator activity	8/569	49/8341	0.016784063
GO:0004674	protein serine/threonine kinase activity	9/569	61/8341	0.021577792
GO:0098772	molecular function regulator	36/569	374/8341	0.021824535
GO:0005085	guanyl-nucleotide exchange factor activity	13/569	103/8341	0.02242232
GO:0031625	ubiquitin protein ligase binding	3/569	10/8341	0.026400465
GO:0044389	ubiquitin-like protein ligase binding	3/569	10/8341	0.026400465
GO:0140098	catalytic activity, acting on RNA	13/569	106/8341	0.027710105
GO:0000287	magnesium ion binding	5/569	26/8341	0.02888362
GO:0008194	UDP-glycosyltransferase activity	5/569	26/8341	0.02888362
GO:0060589	nucleoside-triphosphatase regulator activity	8/569	55/8341	0.031762683
GO:0001871	pattern binding	3/569	11/8341	0.034499233
GO:0030247	polysaccharide binding	3/569	11/8341	0.034499233
