# Supplementary material for: Comparative transcriptome analysis reveals the patterns of gene expression in different venison cuts of sika deer (Cervus nippon)
Source: Anim Biosci. 2025 May 12;38(11):2324–35. doi: 10.5713/ab.25.0044 (PMC12580950; doi:10.5713/ab.25.0044)
Supplement: Supplementary file 11 [file ab-25-0044-supplementary-11.pdf]

Supplement 11. The KEGG enrichment results of DEGs between LD and GM

KEGGID	Description	GeneRatio	BgRatio	pvalue
bta05320	Autoimmune thyroid disease	5/155	44/7990	0.001519326
bta04010	MAPK signaling pathway	14/155	307/7990	0.002488893
bta05144	Malaria	5/155	50/7990	0.002700496
bta04145	Phagosome	9/155	167/7990	0.00509602
bta04514	Cell adhesion molecules	9/155	167/7990	0.00509602
bta04668	TNF signaling pathway	7/155	111/7990	0.005692348
bta05332	Graft-versus-host disease	4/155	39/7990	0.006610261
bta04512	ECM-receptor interaction	6/155	87/7990	0.006714521
bta05330	Allograft rejection	4/155	40/7990	0.007235883
bta05150	Staphylococcus aureus infection	5/155	68/7990	0.010061905
bta04610	Complement and coagulation cascades	5/155	73/7990	0.0134266
bta04940	Type I diabetes mellitus	4/155	49/7990	0.014672457
bta00350	Tyrosine metabolism	3/155	28/7990	0.01644406
bta04612	Antigen processing and presentation	5/155	78/7990	0.017479316
bta05202	Transcriptional misregulation in cancer	9/155	206/7990	0.018629874
bta04064	NF-kappa B signaling pathway	6/155	112/7990	0.021491244
bta04151	PI3K-Akt signaling pathway	13/155	362/7990	0.023380317
bta05142	Chagas disease	6/155	115/7990	0.024120124
bta05205	Proteoglycans in cancer	9/155	228/7990	0.033094462
bta04510	Focal adhesion	8/155	198/7990	0.038578525
bta04936	Alcoholic liver disease	6/155	131/7990	0.041778183
