# Supplementary material for: Comparative transcriptome analysis reveals the patterns of gene expression in different venison cuts of sika deer (Cervus nippon)
Source: Anim Biosci. 2025 May 12;38(11):2324–35. doi: 10.5713/ab.25.0044 (PMC12580950; doi:10.5713/ab.25.0044)
Supplement: Supplementary file 12 [file ab-25-0044-supplementary-12.pdf]

Supplement 12. The GO enrichment results of DEGs between LD and GM

GOID	Description	GeneRatio	BgRatio	pvalue
GO:0030203	glycosaminoglycan metabolic process	3/114	11/5198	0.001491029
GO:0006022	aminoglycan metabolic process	3/114	12/5198	0.001956422
GO:0005576	extracellular region	9/69	216/3220	0.038900092
GO:0005886	plasma membrane	4/69	61/3220	0.040358273
GO:0030414	peptidase inhibitor activity	6/185	54/8322	0.001184749
GO:0061134	peptidase regulator activity	6/185	54/8322	0.001184749
GO:0004857	enzyme inhibitor activity	6/185	67/8322	0.003609283
GO:0030234	enzyme regulator activity	8/185	138/8322	0.011688265
GO:0004867	serine-type endopeptidase inhibitor activity	3/185	23/8322	0.013799636
GO:0051087	chaperone binding	2/185	10/8322	0.019669721
GO:0098772	molecular function regulator	15/185	375/8322	0.019724729
GO:0004252	serine-type endopeptidase activity	6/185	107/8322	0.031710375
GO:0051082	unfolded protein binding	3/185	34/8322	0.039060841
GO:0008236	serine-type peptidase activity	6/185	118/8322	0.047501845
GO:0017171	serine hydrolase activity	6/185	118/8322	0.047501845
