# Supplementary material for: Comparative transcriptome analysis reveals the patterns of gene expression in different venison cuts of sika deer (Cervus nippon)
Source: Anim Biosci. 2025 May 12;38(11):2324–35. doi: 10.5713/ab.25.0044 (PMC12580950; doi:10.5713/ab.25.0044)
Supplement: Supplementary file 13 [file ab-25-0044-supplementary-13.pdf]

Supplement 13. The KEGG enrichment results of DEGs between QF and T

KEGGID	Description	GeneRatio	BgRatio	pvalue
bta04922	Glucagon signaling pathway	25/833	114/8004	0.000221413
bta03050	Proteasome	14/833	52/8004	0.000634086
bta03018	RNA degradation	19/833	86/8004	0.001109275
bta00010	Glycolysis / Gluconeogenesis	17/833	74/8004	0.001262434
bta00500	Starch and sucrose metabolism	8/833	28/8004	0.006212155
bta04970	Salivary secretion	17/833	94/8004	0.016052519
bta00531	Glycosaminoglycan degradation	6/833	22/8004	0.021655636
bta04066	HIF-1 signaling pathway	21/833	130/8004	0.026920567
bta00620	Pyruvate metabolism	10/833	49/8004	0.027134009
bta04140	Autophagy - animal	28/833	186/8004	0.028401028
bta04120	Ubiquitin mediated proteolysis	25/833	163/8004	0.0302106
bta00051	Fructose and mannose metabolism	7/833	30/8004	0.031070203
bta05017	Spinocerebellar ataxia	24/833	157/8004	0.034385114
bta04218	Cellular senescence	26/833	175/8004	0.038919481
bta04340	Hedgehog signaling pathway	10/833	52/8004	0.03940996
bta00430	Taurine and hypotaurine metabolism	5/833	19/8004	0.040689002
bta01200	Carbon metabolism	20/833	130/8004	0.047482557
