# Supplementary material for: Comparative transcriptome analysis reveals the patterns of gene expression in different venison cuts of sika deer (Cervus nippon)
Source: Anim Biosci. 2025 May 12;38(11):2324–35. doi: 10.5713/ab.25.0044 (PMC12580950; doi:10.5713/ab.25.0044)
Supplement: Supplementary file 14 [file ab-25-0044-supplementary-14.pdf]

Supplement 14. The GO enrichment results of DEGs between QF and T

GOID	Description	GeneRatio	BgRatio	pvalue
GO:0005975	carbohydrate metabolic process	27/530	115/5202	2.25E-05
GO:0006090	pyruvate metabolic process	7/530	11/5202	2.51E-05
GO:0006096	glycolytic process	7/530	11/5202	2.51E-05
GO:0006165	nucleoside diphosphate phosphorylation	7/530	11/5202	2.51E-05
GO:0006757	ATP generation from ADP	7/530	11/5202	2.51E-05
GO:0009132	nucleoside diphosphate metabolic process	7/530	11/5202	2.51E-05
GO:0009135	purine nucleoside diphosphate metabolic process	7/530	11/5202	2.51E-05
GO:0009179	purine ribonucleoside diphosphate metabolic process	7/530	11/5202	2.51E-05
GO:0009185	ribonucleoside diphosphate metabolic process	7/530	11/5202	2.51E-05
GO:0016052	carbohydrate catabolic process	7/530	11/5202	2.51E-05
GO:0042866	pyruvate biosynthetic process	7/530	11/5202	2.51E-05
GO:0046031	ADP metabolic process	7/530	11/5202	2.51E-05
GO:0046939	nucleotide phosphorylation	7/530	11/5202	2.51E-05
GO:0019359	nicotinamide nucleotide biosynthetic process	8/530	16/5202	6.76E-05
GO:0019363	pyridine nucleotide biosynthetic process	8/530	17/5202	0.000116369
GO:0072525	pyridine-containing compound biosynthetic process	8/530	17/5202	0.000116369
GO:0044248	cellular catabolic process	25/530	116/5202	0.000198257
GO:0019362	pyridine nucleotide metabolic process	8/530	19/5202	0.000300688
GO:0046496	nicotinamide nucleotide metabolic process	8/530	19/5202	0.000300688
GO:0072524	pyridine-containing compound metabolic process	8/530	19/5202	0.000300688
GO:0019220	regulation of phosphate metabolic process	7/530	15/5202	0.000339661
GO:0051174	regulation of phosphorus metabolic process	7/530	15/5202	0.000339661
GO:0072330	monocarboxylic acid biosynthetic process	7/530	15/5202	0.000339661
GO:0009166	nucleotide catabolic process	7/530	16/5202	0.000551271
GO:0006733	oxidoreduction coenzyme metabolic process	8/530	21/5202	0.000673134
GO:1901575	organic substance catabolic process	25/530	127/5202	0.000842537
GO:0009056	catabolic process	26/530	135/5202	0.000940817
GO:0006091	generation of precursor metabolites and energy	9/530	27/5202	0.000962588
GO:0032268	regulation of cellular protein metabolic process	7/530	18/5202	0.00127873
GO:1901292	nucleoside phosphate catabolic process	7/530	18/5202	0.00127873
GO:0034655	nucleobase-containing compound catabolic process	9/530	28/5202	0.001292709
GO:0032787	monocarboxylic acid metabolic process	8/530	23/5202	0.001349329
GO:0031399	regulation of protein modification process	5/530	10/5202	0.001756273
GO:0016053	organic acid biosynthetic process	7/530	19/5202	0.001849255
GO:0034404	nucleobase-containing small molecule biosynthetic process	7/530	19/5202	0.001849255
GO:0046394	carboxylic acid biosynthetic process	7/530	19/5202	0.001849255
GO:0051246	regulation of protein metabolic process	7/530	19/5202	0.001849255
GO:0044270	cellular nitrogen compound catabolic process	10/530	35/5202	0.001927546
GO:0046700	heterocycle catabolic process	10/530	35/5202	0.001927546
GO:0009152	purine ribonucleotide biosynthetic process	11/530	41/5202	0.002030729
GO:0009260	ribonucleotide biosynthetic process	11/530	41/5202	0.002030729
GO:0046390	ribose phosphate biosynthetic process	11/530	41/5202	0.002030729

GO:0019439	aromatic compound catabolic process	10/530	36/5202	0.002430936
GO:1901361	organic cyclic compound catabolic process	10/530	36/5202	0.002430936
GO:0006754	ATP biosynthetic process	8/530	25/5202	0.002477524
GO:0009142	nucleoside triphosphate biosynthetic process	8/530	25/5202	0.002477524
GO:0009145	purine nucleoside triphosphate biosynthetic process	8/530	25/5202	0.002477524
GO:0009201	ribonucleoside triphosphate biosynthetic process	8/530	25/5202	0.002477524
GO:0009206	purine ribonucleoside triphosphate biosynthetic process	8/530	25/5202	0.002477524
GO:0051186	cofactor metabolic process	13/530	55/5202	0.002853921
GO:0009108	coenzyme biosynthetic process	9/530	31/5202	0.00285745
GO:0019752	carboxylic acid metabolic process	18/530	90/5202	0.003635656
GO:0006164	purine nucleotide biosynthetic process	11/530	44/5202	0.003718949
GO:0006511	ubiquitin-dependent protein catabolic process	11/530	44/5202	0.003718949
GO:0019941	modification-dependent protein catabolic process	11/530	44/5202	0.003718949
GO:0043632	modification-dependent macromolecule catabolic process	11/530	44/5202	0.003718949
GO:0006082	organic acid metabolic process	18/530	91/5202	0.004115672
GO:0043436	oxoacid metabolic process	18/530	91/5202	0.004115672
GO:0009124	nucleoside monophosphate biosynthetic process	8/530	27/5202	0.00423534
GO:0009127	purine nucleoside monophosphate biosynthetic process	8/530	27/5202	0.00423534
GO:0009156	ribonucleoside monophosphate biosynthetic process	8/530	27/5202	0.00423534
GO:0009168	purine ribonucleoside monophosphate biosynthetic process	8/530	27/5202	0.00423534
GO:0072522	purine-containing compound biosynthetic process	11/530	45/5202	0.004481699
GO:0006732	coenzyme metabolic process	9/530	35/5202	0.006919896
GO:0017144	drug metabolic process	12/530	54/5202	0.006942806
GO:0030163	protein catabolic process	13/530	61/5202	0.007315949
GO:0044265	cellular macromolecule catabolic process	14/530	68/5202	0.007530955
GO:0009057	macromolecule catabolic process	15/530	75/5202	0.007618856
GO:0046434	organophosphate catabolic process	7/530	24/5202	0.00809442
GO:0051188	cofactor biosynthetic process	9/530	37/5202	0.010146033
GO:0009150	purine ribonucleotide metabolic process	12/530	57/5202	0.010802383
GO:0009259	ribonucleotide metabolic process	12/530	57/5202	0.010802383
GO:0044257	cellular protein catabolic process	12/530	57/5202	0.010802383
GO:0051603	proteolysis involved in cellular protein catabolic process	12/530	57/5202	0.010802383
GO:0009893	positive regulation of metabolic process	7/530	26/5202	0.012862555
GO:0010604	positive regulation of macromolecule metabolic process	7/530	26/5202	0.012862555
GO:0031325	positive regulation of cellular metabolic process	7/530	26/5202	0.012862555
GO:0051173	positive regulation of nitrogen compound metabolic process	7/530	26/5202	0.012862555
GO:0019693	ribose phosphate metabolic process	12/530	59/5202	0.014177796
GO:0046034	ATP metabolic process	9/530	39/5202	0.014390378
GO:1901565	organonitrogen compound catabolic process	15/530	81/5202	0.015387903
GO:0006163	purine nucleotide metabolic process	12/530	60/5202	0.016140396
GO:0009144	purine nucleoside triphosphate metabolic process	9/530	40/5202	0.016946647
GO:0009199	ribonucleoside triphosphate metabolic process	9/530	40/5202	0.016946647
GO:0009205	purine ribonucleoside triphosphate metabolic process	9/530	40/5202	0.016946647
GO:0072521	purine-containing compound metabolic process	12/530	61/5202	0.018301485

GO:0043244	regulation of protein complex disassembly	4/530	11/5202	0.019623876
GO:0009123	nucleoside monophosphate metabolic process	9/530	41/5202	0.019819479
GO:0009126	purine nucleoside monophosphate metabolic process	9/530	41/5202	0.019819479
GO:0009161	ribonucleoside monophosphate metabolic process	9/530	41/5202	0.019819479
GO:0009167	purine ribonucleoside monophosphate metabolic process	9/530	41/5202	0.019819479
GO:0009141	nucleoside triphosphate metabolic process	9/530	42/5202	0.023028438
GO:0006413	translational initiation	5/530	17/5202	0.023622881
GO:0032984	protein-containing complex disassembly	5/530	17/5202	0.023622881
GO:0043624	cellular protein complex disassembly	5/530	17/5202	0.023622881
GO:0051336	regulation of hydrolase activity	6/530	23/5202	0.024283194
GO:0050790	regulation of catalytic activity	7/530	30/5202	0.027889997
GO:0065009	regulation of molecular function	7/530	30/5202	0.027889997
GO:0022411	cellular component disassembly	5/530	18/5202	0.030045911
GO:0035556	intracellular signal transduction	35/530	251/5202	0.032021808
GO:0006913	nucleocytoplasmic transport	4/530	13/5202	0.036090934
GO:0051169	nuclear transport	4/530	13/5202	0.036090934
GO:1901137	carbohydrate derivative biosynthetic process	18/530	114/5202	0.03858662
GO:0051641	cellular localization	20/530	130/5202	0.038672064
GO:0009165	nucleotide biosynthetic process	12/530	69/5202	0.044034348
GO:1901293	nucleoside phosphate biosynthetic process	12/530	69/5202	0.044034348
GO:0048519	negative regulation of biological process	11/530	62/5202	0.046340411
GO:0046907	intracellular transport	17/530	109/5202	0.048179504
GO:1901135	carbohydrate derivative metabolic process	23/530	158/5202	0.048712914
GO:0015629	actin cytoskeleton	11/294	52/3238	0.00584219
GO:0030133	transport vesicle	4/294	11/3238	0.013138744
GO:0044421	extracellular region part	9/294	45/3238	0.017577168
GO:0019773	proteasome core complex, alpha-subunit complex	4/294	12/3238	0.018325355
GO:0044430	cytoskeletal part	14/294	88/3238	0.025410768
GO:0005856	cytoskeleton	16/294	105/3238	0.025537607
GO:1905368	peptidase complex	7/294	36/3238	0.039766595
GO:0005798	Golgi-associated vesicle	4/294	15/3238	0.040689941
GO:0012506	vesicle membrane	4/294	15/3238	0.040689941
GO:0030120	vesicle coat	4/294	15/3238	0.040689941
GO:0030135	coated vesicle	4/294	15/3238	0.040689941
GO:0030659	cytoplasmic vesicle membrane	4/294	15/3238	0.040689941
GO:0030660	Golgi-associated vesicle membrane	4/294	15/3238	0.040689941
GO:0030662	coated vesicle membrane	4/294	15/3238	0.040689941
GO:0005615	extracellular space	6/294	29/3238	0.04230196
GO:0016459	myosin complex	7/294	37/3238	0.045388597
GO:1902494	catalytic complex	17/294	122/3238	0.046789769
GO:0003723	RNA binding	35/900	208/8346	0.004850376
GO:0016829	lyase activity	13/900	57/8346	0.006579328
GO:0051287	NAD binding	7/900	25/8346	0.013955302
GO:0008135	translation factor activity, RNA binding	8/900	31/8346	0.014615627

GO:0004540	ribonuclease activity	6/900	22/8346	0.025398295
GO:0008081	phosphoric diester hydrolase activity	10/900	48/8346	0.029578393
GO:0003743	translation initiation factor activity	5/900	18/8346	0.037429974
GO:0004435	phosphatidylinositol phospholipase C activity	4/900	13/8346	0.043399404
GO:0004559	alpha-mannosidase activity	4/900	13/8346	0.043399404
GO:0004629	phospholipase C activity	4/900	13/8346	0.043399404
GO:0015923	mannosidase activity	4/900	13/8346	0.043399404
