# Supplementary material for: Comparative transcriptome analysis reveals the patterns of gene expression in different venison cuts of sika deer (Cervus nippon)
Source: Anim Biosci. 2025 May 12;38(11):2324–35. doi: 10.5713/ab.25.0044 (PMC12580950; doi:10.5713/ab.25.0044)
Supplement: Supplementary file 15 [file ab-25-0044-supplementary-15.pdf]

Supplement 15. The KEGG enrichment results of DEGs between QF and IM

KEGGID	Description	GeneRatio	BgRatio	pvalue
bta04928	Parathyroid hormone synthesis, secretion and action	19/567	111/8004	0.000261316
bta00051	Fructose and mannose metabolism	8/567	30/8004	0.000874507
bta04360	Axon guidance	24/567	197/8004	0.006178473
bta04510	Focal adhesion	24/567	202/8004	0.00839302
bta04610	Complement and coagulation cascades	12/567	79/8004	0.0091847
bta04512	ECM-receptor interaction	13/567	89/8004	0.009488174
bta00500	Starch and sucrose metabolism	6/567	28/8004	0.012110047
bta04810	Regulation of actin cytoskeleton	26/567	233/8004	0.013642954
bta04814	Motor proteins	24/567	214/8004	0.016413551
bta04550	Signaling pathways regulating pluripotency of stem cells	17/567	139/8004	0.018678698
bta05207	Chemical carcinogenesis - receptor activation	21/567	183/8004	0.018883405
bta05171	Coronavirus disease - COVID-19	40/567	406/8004	0.019912322
bta04261	Adrenergic signaling in cardiomyocytes	19/567	165/8004	0.023949803
bta04922	Glucagon signaling pathway	14/567	112/8004	0.026251535
bta05143	African trypanosomiasis	6/567	33/8004	0.026399193
bta05230	Central carbon metabolism in cancer	11/567	81/8004	0.026844806
bta03050	Proteasome	8/567	52/8004	0.028587143
bta04152	AMPK signaling pathway	16/567	136/8004	0.030508066
bta05146	Amoebiasis	13/567	104/8004	0.031496843
bta04930	Type II diabetes mellitus	7/567	44/8004	0.033474489
bta04310	Wnt signaling pathway	19/567	172/8004	0.034921266
bta03010	Ribosome	32/567	328/8004	0.039169807
bta04911	Insulin secretion	11/567	86/8004	0.039512391
bta04918	Thyroid hormone synthesis	10/567	76/8004	0.040924866
bta00650	Butanoate metabolism	4/567	19/8004	0.041170428
bta04923	Regulation of lipolysis in adipocytes	8/567	57/8004	0.04621703
