# Supplementary material for: Comparative transcriptome analysis reveals the patterns of gene expression in different venison cuts of sika deer (Cervus nippon)
Source: Anim Biosci. 2025 May 12;38(11):2324–35. doi: 10.5713/ab.25.0044 (PMC12580950; doi:10.5713/ab.25.0044)
Supplement: Supplementary file 16 [file ab-25-0044-supplementary-16.pdf]

Supplement 16. The GO enrichment results of DEGs between QF and IM

GOID	Description	GeneRatio	BgRatio	pvalue
GO:0007167	enzyme linked receptor protein signaling pathway	4/362	16/5203	0.021470448
GO:0019359	nicotinamide nucleotide biosynthetic process	4/362	17/5203	0.026575524
GO:0019363	pyridine nucleotide biosynthetic process	4/362	18/5203	0.032346614
GO:0072525	pyridine-containing compound biosynthetic process	4/362	18/5203	0.032346614
GO:0035556	intracellular signal transduction	25/362	252/5203	0.043573491
GO:0006090	pyruvate metabolic process	3/362	12/5203	0.045886481
GO:0006096	glycolytic process	3/362	12/5203	0.045886481
GO:0006165	nucleoside diphosphate phosphorylation	3/362	12/5203	0.045886481
GO:0006757	ATP generation from ADP	3/362	12/5203	0.045886481
GO:0009132	nucleoside diphosphate metabolic process	3/362	12/5203	0.045886481
GO:0009135	purine nucleoside diphosphate metabolic process	3/362	12/5203	0.045886481
GO:0009179	purine ribonucleoside diphosphate metabolic process	3/362	12/5203	0.045886481
GO:0009185	ribonucleoside diphosphate metabolic process	3/362	12/5203	0.045886481
GO:0042866	pyruvate biosynthetic process	3/362	12/5203	0.045886481
GO:0046031	ADP metabolic process	3/362	12/5203	0.045886481
GO:0046939	nucleotide phosphorylation	3/362	12/5203	0.045886481
GO:0019362	pyridine nucleotide metabolic process	4/362	20/5203	0.045921036
GO:0046496	nicotinamide nucleotide metabolic process	4/362	20/5203	0.045921036
GO:0072524	pyridine-containing compound metabolic process	4/362	20/5203	0.045921036
GO:0005576	extracellular region	24/225	215/3245	0.011941389
GO:0099080	supramolecular complex	4/225	16/3245	0.021110411
GO:0099081	supramolecular polymer	4/225	16/3245	0.021110411
GO:0099512	supramolecular fiber	4/225	16/3245	0.021110411
GO:0015629	actin cytoskeleton	8/225	53/3245	0.027655698
GO:0005856	cytoskeleton	13/225	105/3245	0.027684892
GO:0044430	cytoskeletal part	11/225	88/3245	0.038706353
GO:0016459	myosin complex	6/225	37/3245	0.039439479
GO:0044454	nuclear chromosome part	3/225	12/3245	0.04537118
GO:0015291	secondary active transmembrane transporter activity	8/597	40/8359	0.006451824
GO:0005509	calcium ion binding	32/597	304/8359	0.016859734
GO:0005198	structural molecule activity	30/597	283/8359	0.018542289
GO:0005102	signaling receptor binding	22/597	193/8359	0.019340028
GO:0004180	carboxypeptidase activity	5/597	23/8359	0.020943543
GO:0004674	protein serine/threonine kinase activity	9/597	61/8359	0.028067832
GO:0019199	transmembrane receptor protein kinase activity	4/597	17/8359	0.02903513
GO:0008237	metallopeptidase activity	13/597	103/8359	0.031160842
GO:0005201	extracellular matrix structural constituent	4/597	19/8359	0.042247848
GO:0004190	aspartic-type endopeptidase activity	3/597	12/8359	0.049093134
GO:0070001	aspartic-type peptidase activity	3/597	12/8359	0.049093134
GO:0004181	metallocarboxypeptidase activity	4/597	20/8359	0.049930303
GO:0008235	metalloexopeptidase activity	4/597	20/8359	0.049930303
