# Supplementary material for: Comparative transcriptome analysis reveals the patterns of gene expression in different venison cuts of sika deer (Cervus nippon)
Source: Anim Biosci. 2025 May 12;38(11):2324–35. doi: 10.5713/ab.25.0044 (PMC12580950; doi:10.5713/ab.25.0044)
Supplement: Supplementary file 17 [file ab-25-0044-supplementary-17.pdf]

Supplement 17. The KEGG enrichment results of DEGs between QF and BB

KEGGID	Description	GeneRatio	BgRatio	pvalue
bta05132	Salmonella infection	19/254	285/7996	0.0017702
bta05216	Thyroid cancer	6/254	46/7996	0.003122924
bta04660	T cell receptor signaling pathway	10/254	119/7996	0.004465104
bta04010	MAPK signaling pathway	18/254	301/7996	0.007260276
bta04659	Th17 cell differentiation	9/254	116/7996	0.011337697
bta04550	Signaling pathways regulating pluripotency of stem cells	10/254	137/7996	0.011731065
bta04380	Osteoclast differentiation	9/254	117/7996	0.011952876
bta05135	Yersinia infection	10/254	141/7996	0.014156766
bta04914	Progesterone-mediated oocyte maturation	8/254	105/7996	0.018287647
bta04722	Neurotrophin signaling pathway	9/254	128/7996	0.020468215
bta05218	Melanoma	6/254	69/7996	0.021877442
bta04520	Adherens junction	8/254	109/7996	0.022384387
bta03008	Ribosome biogenesis in eukaryotes	7/254	89/7996	0.022711321
bta05235	PD-L1 expression and PD-1 checkpoint pathway in cancer	7/254	90/7996	0.023984547
bta04068	FoxO signaling pathway	9/254	133/7996	0.025521977
bta04510	Focal adhesion	12/254	201/7996	0.026322238
bta05230	Central carbon metabolism in cancer	6/254	80/7996	0.041250806
bta04613	Neutrophil extracellular trap formation	10/254	169/7996	0.04268429
bta04664	Fc epsilon RI signaling pathway	5/254	63/7996	0.049151138
