# Supplementary material for: Comparative transcriptome analysis reveals the patterns of gene expression in different venison cuts of sika deer (Cervus nippon)
Source: Anim Biosci. 2025 May 12;38(11):2324–35. doi: 10.5713/ab.25.0044 (PMC12580950; doi:10.5713/ab.25.0044)
Supplement: Supplementary file 18 [file ab-25-0044-supplementary-18.pdf]

Supplement 18. The KEGG enrichment results of DEGs between QF and BB

GOID	Description	GeneRatio	BgRatio	pvalue
GO:0006468	protein phosphorylation	31/181	452/5201	0.00015769
GO:0016310	phosphorylation	32/181	481/5201	0.000214899
GO:0007010	cytoskeleton organization	7/181	71/5201	0.011185963
GO:0019220	regulation of phosphate metabolic process	3/181	15/5201	0.013850017
GO:0051174	regulation of phosphorus metabolic process	3/181	15/5201	0.013850017
GO:0051641	cellular localization	10/181	130/5201	0.014600528
GO:0048519	negative regulation of biological process	6/181	62/5201	0.020001517
GO:0032268	regulation of cellular protein metabolic process	3/181	18/5201	0.023013208
GO:0016311	dephosphorylation	7/181	83/5201	0.024698535
GO:0006887	exocytosis	3/181	19/5201	0.026644058
GO:0032940	secretion by cell	3/181	19/5201	0.026644058
GO:0051246	regulation of protein metabolic process	3/181	19/5201	0.026644058
GO:0046907	intracellular transport	8/181	109/5201	0.035285756
GO:0051649	establishment of localization in cell	8/181	110/5201	0.036965239
GO:0048523	negative regulation of cellular process	5/181	55/5201	0.041303809
GO:0006886	intracellular protein transport	7/181	93/5201	0.042306197
GO:0000226	microtubule cytoskeleton organization	3/181	23/5201	0.044020829
GO:0031399	regulation of protein modification process	2/181	10/5201	0.045102779
GO:1902904	negative regulation of supramolecular fiber organization	2/181	10/5201	0.045102779
GO:0009892	negative regulation of metabolic process	3/181	24/5201	0.049061718
GO:0051493	regulation of cytoskeleton organization	3/181	24/5201	0.049061718
GO:0032993	protein-DNA complex	8/92	53/3244	9.58E-05
GO:0000786	nucleosome	7/92	52/3244	0.000554167
GO:0044815	DNA packaging complex	7/92	53/3244	0.000623922
GO:0000785	chromatin	7/92	62/3244	0.001617449
GO:0044427	chromosomal part	8/92	93/3244	0.004344343
GO:0005694	chromosome	8/92	104/3244	0.008517709
GO:0030117	membrane coat	3/92	26/3244	0.035820913
GO:0048475	coated membrane	3/92	26/3244	0.035820913
GO:0016591	DNA-directed RNA polymerase II, holoenzyme	2/92	12/3244	0.043652782
GO:0004672	protein kinase activity	32/283	457/8349	6.88E-05
GO:0004674	protein serine/threonine kinase activity	9/283	61/8349	0.000191727
GO:0030695	GTPase regulator activity	5/283	49/8349	0.024398654
GO:0046982	protein heterodimerization activity	6/283	72/8349	0.034742136
GO:0060589	nucleoside-triphosphatase regulator activity	5/283	55/8349	0.037843483
GO:0005096	GTPase activator activity	4/283	38/8349	0.038639693
