# Supplementary material for: Comparative transcriptome analysis reveals the patterns of gene expression in different venison cuts of sika deer (Cervus nippon)
Source: Anim Biosci. 2025 May 12;38(11):2324–35. doi: 10.5713/ab.25.0044 (PMC12580950; doi:10.5713/ab.25.0044)
Supplement: Supplementary file 19 [file ab-25-0044-supplementary-19.pdf]

Supplement 19. The KEGG enrichment results of DEGs between QF and GM

KEGGID	Description	GeneRatio	BgRatio	pvalue
bta04610	Complement and coagulation cascades	7/140	76/7983	0.000349993
bta04060	Cytokine-cytokine receptor interaction	12/140	227/7983	0.000613893
bta05144	Malaria	5/140	49/7983	0.001584268
bta04061	Viral protein interaction with cytokine and cytokine receptor	6/140	75/7983	0.001950033
bta04062	Chemokine signaling pathway	9/140	173/7983	0.003302857
bta04630	JAK-STAT signaling pathway	7/140	130/7983	0.007742337
bta05206	MicroRNAs in cancer	8/140	176/7983	0.012067756
bta04742	Taste transduction	5/140	93/7983	0.023451126
bta04145	Phagosome	7/140	165/7983	0.025781276
bta00565	Ether lipid metabolism	3/140	40/7983	0.032526033
bta00430	Taurine and hypotaurine metabolism	2/140	17/7983	0.034962955
bta04010	MAPK signaling pathway	10/140	305/7983	0.04154776
