# Supplementary material for: Comparative transcriptome analysis reveals the patterns of gene expression in different venison cuts of sika deer (Cervus nippon)
Source: Anim Biosci. 2025 May 12;38(11):2324–35. doi: 10.5713/ab.25.0044 (PMC12580950; doi:10.5713/ab.25.0044)
Supplement: Supplementary file 20 [file ab-25-0044-supplementary-20.pdf]

Supplement 20. The GO enrichment results of DEGs between QF and GM

GOID	Description	GeneRatio	BgRatio	pvalue
GO:0030001	metal ion transport	5/77	79/5192	0.006036028
GO:0032504	multicellular organism reproduction	2/77	10/5192	0.009045337
GO:0006955	immune response	5/77	90/5192	0.010378907
GO:0002376	immune system process	5/77	94/5192	0.012384265
GO:0019953	sexual reproduction	2/77	12/5192	0.013014442
GO:0044703	multi-organism reproductive process	2/77	12/5192	0.013014442
GO:0051704	multi-organism process	2/77	20/5192	0.034713595
GO:0000003	reproduction	2/77	22/5192	0.041411532
GO:0006814	sodium ion transport	2/77	22/5192	0.041411532
GO:0022414	reproductive process	2/77	22/5192	0.041411532
GO:0034702	ion channel complex	3/45	18/3224	0.001791473
GO:0034703	cation channel complex	3/45	18/3224	0.001791473
GO:0005576	extracellular region	9/45	216/3224	0.002439734
GO:1902495	transmembrane transporter complex	3/45	22/3224	0.003251543
GO:1990351	transporter complex	3/45	22/3224	0.003251543
GO:0005886	plasma membrane	4/45	60/3224	0.009142199
GO:0071944	cell periphery	4/45	69/3224	0.014798507
GO:0098797	plasma membrane protein complex	3/45	45/3224	0.023971646
GO:0005887	integral component of plasma membrane	2/45	18/3224	0.025294384
GO:0044459	plasma membrane part	3/45	53/3224	0.036642578
GO:0031226	intrinsic component of plasma membrane	2/45	22/3224	0.036869695
GO:0016709	oxidoreductase activity, acting on paired donors, with incorporation or reduction of molecular oxygen, NAD(P)H as one donor, and incorporation of one atom of oxygen	3/138	11/8328	0.000666425
GO:0005125	cytokine activity	4/138	41/8328	0.00454539
GO:0030545	receptor regulator activity	7/138	130/8328	0.005736899
GO:0048018	receptor ligand activity	7/138	130/8328	0.005736899
GO:0016705	oxidoreductase activity, acting on paired donors, with incorporation or reduction of molecular oxygen	5/138	71/8328	0.006304364
GO:0005261	cation channel activity	4/138	46/8328	0.006873096
GO:0005044	scavenger receptor activity	3/138	25/8328	0.007841556
GO:0038024	cargo receptor activity	3/138	25/8328	0.007841556
GO:0004497	monooxygenase activity	3/138	26/8328	0.008758343
GO:0098772	molecular function regulator	13/138	376/8328	0.009356986
GO:0046873	metal ion transmembrane transporter activity	5/138	88/8328	0.015188446
GO:0005102	signaling receptor binding	8/138	195/8328	0.015594198
GO:0005272	sodium channel activity	2/138	15/8328	0.024855723
GO:0050661	NADP binding	2/138	15/8328	0.024855723
GO:0005507	copper ion binding	2/138	16/8328	0.028102464
GO:0050660	flavin adenine dinucleotide binding	3/138	40/8328	0.028150745
GO:0140097	catalytic activity, acting on DNA	3/138	40/8328	0.028150745
GO:0004866	endopeptidase inhibitor activity	3/138	44/8328	0.035985895
GO:0061135	endopeptidase regulator activity	3/138	44/8328	0.035985895
