# Supplementary material for: Comparative transcriptome analysis reveals the patterns of gene expression in different venison cuts of sika deer (Cervus nippon)
Source: Anim Biosci. 2025 May 12;38(11):2324–35. doi: 10.5713/ab.25.0044 (PMC12580950; doi:10.5713/ab.25.0044)
Supplement: Supplementary file 21 [file ab-25-0044-supplementary-21.pdf]

Supplement 21. The KEGG enrichment results of DEGs between T and IM

KEGGID	Description	GeneRatio	BgRatio	pvalue
bta05144	Malaria	5/203	49/8033	0.007588717
bta04152	AMPK signaling pathway	9/203	137/8033	0.007773187
bta04610	Complement and coagulation cascades	6/203	76/8033	0.012196469
bta04660	T cell receptor signaling pathway	7/203	119/8033	0.030850728
bta04974	Protein digestion and absorption	6/203	96/8033	0.034302527
bta05204	Chemical carcinogenesis - DNA adducts	3/203	29/8033	0.035866519
bta04080	Neuroactive ligand-receptor interaction	13/203	305/8033	0.045164651
bta00562	Inositol phosphate metabolism	5/203	78/8033	0.046958233
