# Supplementary material for: Comparative transcriptome analysis reveals the patterns of gene expression in different venison cuts of sika deer (Cervus nippon)
Source: Anim Biosci. 2025 May 12;38(11):2324–35. doi: 10.5713/ab.25.0044 (PMC12580950; doi:10.5713/ab.25.0044)
Supplement: Supplementary file 22 [file ab-25-0044-supplementary-22.pdf]

Supplement 22. The GO enrichment results of DEGs between T and IM

GOID	Description	GeneRatio	BgRatio	pvalue
GO:1901615	organic hydroxy compound metabolic process	3/142	16/5229	0.008470363
GO:0006629	lipid metabolic process	9/142	142/5229	0.01459535
GO:0007155	cell adhesion	8/142	121/5229	0.016553912
GO:0022610	biological adhesion	8/142	121/5229	0.016553912
GO:0032502	developmental process	6/142	80/5229	0.020909866
GO:0016055	Wnt signaling pathway	3/142	23/5229	0.023330369
GO:0198738	cell-cell signaling by wnt	3/142	23/5229	0.023330369
GO:1905114	cell surface receptor signaling pathway involved in cell-cell signaling	3/142	23/5229	0.023330369
GO:0009966	regulation of signal transduction	8/142	132/5229	0.026453233
GO:0010646	regulation of cell communication	8/142	132/5229	0.026453233
GO:0023051	regulation of signaling	8/142	133/5229	0.027521494
GO:0046165	alcohol biosynthetic process	2/142	10/5229	0.028565527
GO:1901617	organic hydroxy compound biosynthetic process	2/142	10/5229	0.028565527
GO:0051056	regulation of small GTPase mediated signal transduction	6/142	89/5229	0.033220347
GO:0009116	nucleoside metabolic process	2/142	11/5229	0.034300798
GO:0019751	polyol metabolic process	2/142	11/5229	0.034300798
GO:1901657	glycosyl compound metabolic process	2/142	11/5229	0.034300798
GO:0048583	regulation of response to stimulus	8/142	140/5229	0.035849938
GO:0007186	G-protein-coupled receptor signaling pathway	15/142	335/5229	0.037143348
GO:0007267	cell-cell signaling	3/142	28/5229	0.039132355
GO:0035556	intracellular signal transduction	12/142	253/5229	0.041225253
GO:1902531	regulation of intracellular signal transduction	6/142	95/5229	0.043607076
GO:0005576	extracellular region	16/82	215/3258	6.19E-05
GO:0044421	extracellular region part	4/82	45/3258	0.025393005
GO:0008237	metallopeptidase activity	9/214	101/8390	0.00105715
GO:0004222	metalloendopeptidase activity	7/214	68/8390	0.001649305
GO:0008081	phosphoric diester hydrolase activity	5/214	46/8390	0.006034618
GO:0005179	hormone activity	4/214	34/8390	0.01048009
GO:0005201	extracellular matrix structural constituent	3/214	19/8390	0.011726707
GO:0098772	molecular function regulator	17/214	374/8390	0.014847875
GO:0005261	cation channel activity	4/214	46/8390	0.029134971
GO:0004857	enzyme inhibitor activity	5/214	68/8390	0.029326538
GO:0042578	phosphoric ester hydrolase activity	8/214	147/8390	0.034217413
GO:0004888	transmembrane signaling receptor activity	14/214	322/8390	0.035829869
GO:0005540	hyaluronic acid binding	2/214	12/8390	0.036121041
GO:0004930	G-protein-coupled receptor activity	12/214	270/8390	0.043408752
GO:0038023	signaling receptor activity	14/214	333/8390	0.045402989
GO:0060089	molecular transducer activity	14/214	333/8390	0.045402989
