# Supplementary material for: Comparative transcriptome analysis reveals the patterns of gene expression in different venison cuts of sika deer (Cervus nippon)
Source: Anim Biosci. 2025 May 12;38(11):2324–35. doi: 10.5713/ab.25.0044 (PMC12580950; doi:10.5713/ab.25.0044)
Supplement: Supplementary file 23 [file ab-25-0044-supplementary-23.pdf]

Supplement 23. The KEGG enrichment results of DEGs between T and BB

KEGGID	Description	GeneRatio	BgRatio	pvalue
bta01200	Carbon metabolism	24/467	131/8029	4.11E-07
bta05415	Diabetic cardiomyopathy	33/467	221/8029	4.45E-07
bta00010	Glycolysis / Gluconeogenesis	15/467	72/8029	1.26E-05
bta00020	Citrate cycle (TCA cycle)	11/467	41/8029	1.47E-05
bta00190	Oxidative phosphorylation	22/467	140/8029	1.69E-05
bta04714	Thermogenesis	31/467	244/8029	2.96E-05
bta05208	Chemical carcinogenesis - reactive oxygen species	29/467	226/8029	4.45E-05
bta05020	Prion disease	33/467	283/8029	9.32E-05
bta00620	Pyruvate metabolism	10/467	47/8029	0.000296693
bta04932	Non-alcoholic fatty liver disease	21/467	160/8029	0.000365174
bta04922	Glucagon signaling pathway	16/467	112/8029	0.000709334
bta00030	Pentose phosphate pathway	7/467	28/8029	0.000874489
bta05012	Parkinson disease	31/467	294/8029	0.00088489
bta04066	HIF-1 signaling pathway	17/467	128/8029	0.001134774
bta00051	Fructose and mannose metabolism	7/467	30/8029	0.001358987
bta00640	Propanoate metabolism	8/467	39/8029	0.001532814
bta00785	Lipoic acid metabolism	6/467	23/8029	0.001618426
bta00260	Glycine, serine and threonine metabolism	8/467	41/8029	0.002149274
bta05010	Alzheimer disease	38/467	413/8029	0.003084486
bta04020	Calcium signaling pathway	27/467	279/8029	0.00615695
bta05230	Central carbon metabolism in cancer	11/467	82/8029	0.007563212
bta04064	NF-kappa B signaling pathway	13/467	109/8029	0.010351216
bta04960	Aldosterone-regulated sodium reabsorption	6/467	34/8029	0.012542195
bta01230	Biosynthesis of amino acids	11/467	88/8029	0.012661293
bta01210	2-Oxocarboxylic acid metabolism	6/467	36/8029	0.016474345
bta04022	cGMP-PKG signaling pathway	18/467	182/8029	0.018748316
bta04750	Inflammatory mediator regulation of TRP channels	12/467	110/8029	0.025638578
bta00330	Arginine and proline metabolism	7/467	53/8029	0.032660584
bta04970	Salivary secretion	10/467	91/8029	0.037907009
bta04216	Ferroptosis	6/467	44/8029	0.04051008
bta04115	p53 signaling pathway	9/467	80/8029	0.041814292
bta04213	Longevity regulating pathway - multiple species	8/467	68/8029	0.042610971
bta00520	Amino sugar and nucleotide sugar metabolism	6/467	45/8029	0.04454481
bta05134	Legionellosis	8/467	69/8029	0.045861495
bta05016	Huntington disease	27/467	331/8029	0.046107693
