# Supplementary material for: Comparative transcriptome analysis reveals the patterns of gene expression in different venison cuts of sika deer (Cervus nippon)
Source: Anim Biosci. 2025 May 12;38(11):2324–35. doi: 10.5713/ab.25.0044 (PMC12580950; doi:10.5713/ab.25.0044)
Supplement: Supplementary file 24 [file ab-25-0044-supplementary-24.pdf]

Supplement 24. The GO enrichment results of DEGs between T and BB

GOID	Description	GeneRatio	BgRatio	pvalue
GO:0009150	purine ribonucleotide metabolic process	18/294	57/5226	8.02E-10
GO:0009259	ribonucleotide metabolic process	18/294	57/5226	8.02E-10
GO:0046034	ATP metabolic process	15/294	39/5226	9.28E-10
GO:0009144	purine nucleoside triphosphate metabolic process	15/294	40/5226	1.41E-09
GO:0009199	ribonucleoside triphosphate metabolic process	15/294	40/5226	1.41E-09
GO:0009205	purine ribonucleoside triphosphate metabolic process	15/294	40/5226	1.41E-09
GO:0019693	ribose phosphate metabolic process	18/294	59/5226	1.51E-09
GO:0006163	purine nucleotide metabolic process	18/294	60/5226	2.05E-09
GO:0009123	nucleoside monophosphate metabolic process	15/294	41/5226	2.11E-09
GO:0009126	purine nucleoside monophosphate metabolic process	15/294	41/5226	2.11E-09
GO:0009161	ribonucleoside monophosphate metabolic process	15/294	41/5226	2.11E-09
GO:0009167	purine ribonucleoside monophosphate metabolic process	15/294	41/5226	2.11E-09
GO:0072521	purine-containing compound metabolic process	18/294	61/5226	2.76E-09
GO:0009141	nucleoside triphosphate metabolic process	15/294	42/5226	3.12E-09
GO:0006091	generation of precursor metabolites and energy	12/294	27/5226	6.54E-09
GO:0017144	drug metabolic process	16/294	54/5226	2.03E-08
GO:0009152	purine ribonucleotide biosynthetic process	14/294	41/5226	2.08E-08
GO:0009260	ribonucleotide biosynthetic process	14/294	41/5226	2.08E-08
GO:0046390	ribose phosphate biosynthetic process	14/294	41/5226	2.08E-08
GO:0006754	ATP biosynthetic process	11/294	25/5226	3.26E-08
GO:0009142	nucleoside triphosphate biosynthetic process	11/294	25/5226	3.26E-08
GO:0009145	purine nucleoside triphosphate biosynthetic process	11/294	25/5226	3.26E-08
GO:0009201	ribonucleoside triphosphate biosynthetic process	11/294	25/5226	3.26E-08
GO:0009206	purine ribonucleoside triphosphate biosynthetic process	11/294	25/5226	3.26E-08
GO:0006164	purine nucleotide biosynthetic process	14/294	44/5226	5.82E-08
GO:0072522	purine-containing compound biosynthetic process	14/294	45/5226	8.02E-08
GO:0009124	nucleoside monophosphate biosynthetic process	11/294	27/5226	8.62E-08
GO:0009127	purine nucleoside monophosphate biosynthetic process	11/294	27/5226	8.62E-08
GO:0009156	ribonucleoside monophosphate biosynthetic process	11/294	27/5226	8.62E-08
GO:0009168	purine ribonucleoside monophosphate biosynthetic process	11/294	27/5226	8.62E-08
GO:0009117	nucleotide metabolic process	19/294	93/5226	6.48E-07
GO:0006753	nucleoside phosphate metabolic process	19/294	95/5226	9.16E-07
GO:0055086	nucleobase-containing small molecule metabolic process	20/294	107/5226	1.46E-06
GO:0009165	nucleotide biosynthetic process	15/294	69/5226	4.48E-06
GO:1901293	nucleoside phosphate biosynthetic process	15/294	69/5226	4.48E-06
GO:0072330	monocarboxylic acid biosynthetic process	7/294	15/5226	7.23E-06
GO:0006090	pyruvate metabolic process	6/294	11/5226	1.10E-05
GO:0006096	glycolytic process	6/294	11/5226	1.10E-05
GO:0006165	nucleoside diphosphate phosphorylation	6/294	11/5226	1.10E-05
GO:0006757	ATP generation from ADP	6/294	11/5226	1.10E-05
GO:0009132	nucleoside diphosphate metabolic process	6/294	11/5226	1.10E-05
GO:0009135	purine nucleoside diphosphate metabolic process	6/294	11/5226	1.10E-05

GO:0009179	purine ribonucleoside diphosphate metabolic process	6/294	11/5226	1.10E-05
GO:0009185	ribonucleoside diphosphate metabolic process	6/294	11/5226	1.10E-05
GO:0016052	carbohydrate catabolic process	6/294	11/5226	1.10E-05
GO:0042866	pyruvate biosynthetic process	6/294	11/5226	1.10E-05
GO:0046031	ADP metabolic process	6/294	11/5226	1.10E-05
GO:0046939	nucleotide phosphorylation	6/294	11/5226	1.10E-05
GO:0090407	organophosphate biosynthetic process	18/294	102/5226	1.15E-05
GO:0016053	organic acid biosynthetic process	7/294	19/5226	4.66E-05
GO:0046394	carboxylic acid biosynthetic process	7/294	19/5226	4.66E-05
GO:0019637	organophosphate metabolic process	22/294	157/5226	5.53E-05
GO:0009166	nucleotide catabolic process	6/294	16/5226	0.000149284
GO:0019359	nicotinamide nucleotide biosynthetic process	6/294	16/5226	0.000149284
GO:1901135	carbohydrate derivative metabolic process	21/294	157/5226	0.000161439
GO:0032787	monocarboxylic acid metabolic process	7/294	23/5226	0.000186329
GO:0051186	cofactor metabolic process	11/294	55/5226	0.000191519
GO:0019363	pyridine nucleotide biosynthetic process	6/294	17/5226	0.000219902
GO:0072525	pyridine-containing compound biosynthetic process	6/294	17/5226	0.000219902
GO:0009108	coenzyme biosynthetic process	8/294	31/5226	0.000230677
GO:0022900	electron transport chain	5/294	12/5226	0.000310869
GO:1901292	nucleoside phosphate catabolic process	6/294	18/5226	0.000314411
GO:0019362	pyridine nucleotide metabolic process	6/294	19/5226	0.000438035
GO:0034404	nucleobase-containing small molecule biosynthetic process	6/294	19/5226	0.000438035
GO:0046496	nicotinamide nucleotide metabolic process	6/294	19/5226	0.000438035
GO:0072524	pyridine-containing compound metabolic process	6/294	19/5226	0.000438035
GO:0045333	cellular respiration	5/294	13/5226	0.000482066
GO:0006732	coenzyme metabolic process	8/294	35/5226	0.00056512
GO:0044281	small molecule metabolic process	25/294	224/5226	0.000693248
GO:0034655	nucleobase-containing compound catabolic process	7/294	28/5226	0.000705515
GO:0015980	energy derivation by oxidation of organic compounds	5/294	14/5226	0.000715642
GO:0015985	energy coupled proton transport, down electrochemical gradient	5/294	14/5226	0.000715642
GO:0015986	ATP synthesis coupled proton transport	5/294	14/5226	0.000715642
GO:0006733	oxidoreduction coenzyme metabolic process	6/294	21/5226	0.000796165
GO:0051188	cofactor biosynthetic process	8/294	37/5226	0.000840194
GO:0055114	oxidation-reduction process	34/294	352/5226	0.001143394
GO:1901137	carbohydrate derivative biosynthetic process	15/294	113/5226	0.001506777
GO:0022904	respiratory electron transport chain	4/294	10/5226	0.001572753
GO:0046434	organophosphate catabolic process	6/294	24/5226	0.001711537
GO:0044283	small molecule biosynthetic process	8/294	42/5226	0.00200992
GO:0044270	cellular nitrogen compound catabolic process	7/294	35/5226	0.002854748
GO:0046700	heterocycle catabolic process	7/294	35/5226	0.002854748
GO:0019439	aromatic compound catabolic process	7/294	36/5226	0.003376861
GO:1901361	organic cyclic compound catabolic process	7/294	36/5226	0.003376861
GO:0005743	mitochondrial inner membrane	10/183	41/3264	5.87E-05
GO:0044429	mitochondrial part	14/183	76/3264	5.87E-05

GO:0019866	organelle inner membrane	10/183	42/3264	7.34E-05
GO:0044455	mitochondrial membrane part	10/183	43/3264	9.10E-05
GO:0098800	inner mitochondrial membrane protein complex	8/183	30/3264	0.000168881
GO:0005740	mitochondrial envelope	12/183	67/3264	0.000265914
GO:0031966	mitochondrial membrane	11/183	60/3264	0.000389743
GO:0044422	organelle part	42/183	451/3264	0.000391062
GO:0044446	intracellular organelle part	41/183	448/3264	0.000676209
GO:0098798	mitochondrial protein complex	9/183	45/3264	0.000686937
GO:0031967	organelle envelope	12/183	75/3264	0.000779792
GO:0031975	envelope	12/183	75/3264	0.000779792
GO:0005739	mitochondrion	14/183	97/3264	0.000841216
GO:0031090	organelle membrane	14/183	106/3264	0.002034033
GO:0005746	mitochondrial respiratory chain	4/183	13/3264	0.004583829
GO:0070469	respiratory chain	4/183	13/3264	0.004583829
GO:0015629	actin cytoskeleton	8/183	53/3264	0.008441583
