# Supplementary material for: Comparative transcriptome analysis reveals the patterns of gene expression in different venison cuts of sika deer (Cervus nippon)
Source: Anim Biosci. 2025 May 12;38(11):2324–35. doi: 10.5713/ab.25.0044 (PMC12580950; doi:10.5713/ab.25.0044)
Supplement: Supplementary file 25 [file ab-25-0044-supplementary-25.pdf]

Supplement 25. The KEGG enrichment results of DEGs between IM and BB

KEGGID	Description	GeneRatio	BgRatio	pvalue
bta04610	Complement and coagulation cascades	14/248	75/8018	5.30E-08
bta04640	Hematopoietic cell lineage	10/248	92/8018	0.000518694
bta04020	Calcium signaling pathway	19/248	277/8018	0.000925959
bta05207	Chemical carcinogenesis - receptor activation	14/248	184/8018	0.001655793
bta04310	Wnt signaling pathway	13/248	175/8018	0.002968254
bta04060	Cytokine-cytokine receptor interaction	15/248	225/8018	0.004129147
bta05202	Transcriptional misregulation in cancer	13/248	205/8018	0.010994786
bta04810	Regulation of actin cytoskeleton	14/248	232/8018	0.012757313
bta04630	JAK-STAT signaling pathway	9/248	129/8018	0.018351234
bta05144	Malaria	5/248	50/8018	0.018555578
bta05143	African trypanosomiasis	4/248	34/8018	0.019999414
bta01250	Biosynthesis of nucleotide sugars	4/248	36/8018	0.024217987
bta04152	AMPK signaling pathway	9/248	137/8018	0.025987919
bta05135	Yersinia infection	9/248	141/8018	0.030550172
bta00052	Galactose metabolism	3/248	23/8018	0.032791152
bta04080	Neuroactive ligand-receptor interaction	15/248	298/8018	0.043050991
bta04911	Insulin secretion	6/248	85/8018	0.047465948
bta00520	Amino sugar and nucleotide sugar metabolism	4/248	45/8018	0.049482584
