# Supplementary material for: Comparative transcriptome analysis reveals the patterns of gene expression in different venison cuts of sika deer (Cervus nippon)
Source: Anim Biosci. 2025 May 12;38(11):2324–35. doi: 10.5713/ab.25.0044 (PMC12580950; doi:10.5713/ab.25.0044)
Supplement: Supplementary file 26 [file ab-25-0044-supplementary-26.pdf]

Supplement 26. The GO enrichment results of DEGs between IM and BB

GOID	Description	GeneRatio	BgRatio	pvalue
GO:0051260	protein homooligomerization	5/158	51/5228	0.018145599
GO:0006955	immune response	7/158	90/5228	0.018480285
GO:0002376	immune system process	7/158	94/5228	0.022920657
GO:0051259	protein complex oligomerization	5/158	57/5228	0.028020054
GO:0007155	cell adhesion	8/158	121/5228	0.029325328
GO:0022610	biological adhesion	8/158	121/5228	0.029325328
GO:0006090	pyruvate metabolic process	2/158	11/5228	0.041738036
GO:0006096	glycolytic process	2/158	11/5228	0.041738036
GO:0006165	nucleoside diphosphate phosphorylation	2/158	11/5228	0.041738036
GO:0006757	ATP generation from ADP	2/158	11/5228	0.041738036
GO:0009132	nucleoside diphosphate metabolic process	2/158	11/5228	0.041738036
GO:0009135	purine nucleoside diphosphate metabolic process	2/158	11/5228	0.041738036
GO:0009179	purine ribonucleoside diphosphate metabolic process	2/158	11/5228	0.041738036
GO:0009185	ribonucleoside diphosphate metabolic process	2/158	11/5228	0.041738036
GO:0042866	pyruvate biosynthetic process	2/158	11/5228	0.041738036
GO:0046031	ADP metabolic process	2/158	11/5228	0.041738036
GO:0046939	nucleotide phosphorylation	2/158	11/5228	0.041738036
GO:0007186	G-protein-coupled receptor signaling pathway	16/158	333/5228	0.042736813
GO:0006952	defense response	2/158	12/5228	0.049110822
GO:0016052	carbohydrate catabolic process	2/158	12/5228	0.049110822
GO:0005576	extracellular region	22/112	218/3270	2.59E-06
GO:0044421	extracellular region part	6/112	43/3270	0.003057994
GO:0031012	extracellular matrix	3/112	16/3270	0.015816757
GO:0005044	scavenger receptor activity	6/264	26/8387	0.000124417
GO:0038024	cargo receptor activity	6/264	26/8387	0.000124417
GO:0004222	metalloendopeptidase activity	8/264	68/8387	0.001247944
GO:0030545	receptor regulator activity	11/264	130/8387	0.002584283
GO:0048018	receptor ligand activity	11/264	130/8387	0.002584283
GO:0008237	metallopeptidase activity	9/264	101/8387	0.004419137
GO:0005102	signaling receptor binding	13/264	193/8387	0.007850527
GO:0005216	ion channel activity	12/264	185/8387	0.013809451
GO:0022838	substrate-specific channel activity	12/264	185/8387	0.013809451
GO:0005509	calcium ion binding	17/264	302/8387	0.014507395
GO:0005507	copper ion binding	3/264	17/8387	0.015118663
GO:0098772	molecular function regulator	20/264	378/8387	0.015739815
GO:0004871	signal transducer activity	18/264	336/8387	0.019127118
GO:0015267	channel activity	12/264	196/8387	0.020850081
GO:0022803	passive transmembrane transporter activity	12/264	196/8387	0.020850081
GO:0004175	endopeptidase activity	13/264	224/8387	0.02451794
GO:0020037	heme binding	6/264	74/8387	0.028596501
GO:0038023	signaling receptor activity	17/264	330/8387	0.031189757
GO:0060089	molecular transducer activity	17/264	330/8387	0.031189757

GO:0046906	tetrapyrrole binding	6/264	78/8387	0.03578325
GO:0016614	oxidoreductase activity, acting on CH-OH group of donors	4/264	40/8387	0.036112747
GO:0022836	gated channel activity	6/264	79/8387	0.037745599
GO:0022839	ion gated channel activity	6/264	79/8387	0.037745599
GO:0004930	G-protein-coupled receptor activity	14/264	266/8387	0.041210893
GO:0005125	cytokine activity	4/264	42/8387	0.042145673
GO:0004888	transmembrane signaling receptor activity	16/264	319/8387	0.044122309
GO:0005262	calcium channel activity	2/264	11/8387	0.045013689
