# Supplementary material for: Comparative transcriptome analysis reveals the patterns of gene expression in different venison cuts of sika deer (Cervus nippon)
Source: Anim Biosci. 2025 May 12;38(11):2324–35. doi: 10.5713/ab.25.0044 (PMC12580950; doi:10.5713/ab.25.0044)
Supplement: Supplementary file 27 [file ab-25-0044-supplementary-27.pdf]

Supplement 27. The KEGG enrichment results of DEGs between IM and GM

KEGGID	Description	GeneRatio	BgRatio	pvalue
bta04512	ECM-receptor interaction	15/532	90/8028	0.000761851
bta04610	Complement and coagulation cascades	12/532	74/8028	0.003171685
bta04974	Protein digestion and absorption	14/532	97/8028	0.004517219
bta03013	Nucleocytoplasmic transport	16/532	120/8028	0.005523813
bta05206	MicroRNAs in cancer	20/532	178/8028	0.013846824
bta04151	PI3K-Akt signaling pathway	35/532	363/8028	0.015420144
bta04060	Cytokine-cytokine receptor interaction	23/532	225/8028	0.024625629
bta05340	Primary immunodeficiency	6/532	35/8028	0.025793896
bta04510	Focal adhesion	21/532	203/8028	0.027501327
bta04630	JAK-STAT signaling pathway	14/532	128/8028	0.043429534
