# Supplementary material for: Comparative transcriptome analysis reveals the patterns of gene expression in different venison cuts of sika deer (Cervus nippon)
Source: Anim Biosci. 2025 May 12;38(11):2324–35. doi: 10.5713/ab.25.0044 (PMC12580950; doi:10.5713/ab.25.0044)
Supplement: Supplementary file 28 [file ab-25-0044-supplementary-28.pdf]

Supplement 28. The GO enrichment results of DEGs between IM and GM

GOID	Description	GeneRatio	BgRatio	pvalue
GO:0006508	proteolysis	36/345	363/5225	0.008058346
GO:0032268	regulation of cellular protein metabolic process	4/345	18/5225	0.027301994
GO:0051246	regulation of protein metabolic process	4/345	19/5225	0.032834502
GO:0006468	protein phosphorylation	40/345	457/5225	0.036683034
GO:0016310	phosphorylation	42/345	486/5225	0.03914167
GO:0019953	sexual reproduction	3/345	12/5225	0.040174959
GO:0044703	multi-organism reproductive process	3/345	12/5225	0.040174959
GO:0016055	Wnt signaling pathway	4/345	21/5225	0.045723779
GO:0198738	cell-cell signaling by wnt	4/345	21/5225	0.045723779
GO:1905114	cell surface receptor signaling pathway involved in cell-cell signaling	4/345	21/5225	0.045723779
GO:0006913	nucleocytoplasmic transport	3/345	13/5225	0.049741899
GO:0032011	ARF protein signal transduction	3/345	13/5225	0.049741899
GO:0032012	regulation of ARF protein signal transduction	3/345	13/5225	0.049741899
GO:0051169	nuclear transport	3/345	13/5225	0.049741899
GO:0005576	extracellular region	28/207	215/3248	0.000157065
GO:0099080	supramolecular complex	5/207	18/3248	0.004329785
GO:0099081	supramolecular polymer	5/207	18/3248	0.004329785
GO:0099512	supramolecular fiber	5/207	18/3248	0.004329785
GO:0044421	extracellular region part	7/207	42/3248	0.015387179
GO:0031012	extracellular matrix	4/207	16/3248	0.015887224
GO:0016591	DNA-directed RNA polymerase II, holoenzyme	3/207	12/3248	0.036578398
GO:0004180	carboxypeptidase activity	5/606	23/8384	0.021948268
GO:0005507	copper ion binding	4/606	17/8384	0.030185827
GO:0008237	metallopeptidase activity	13/606	103/8384	0.033886804
GO:0005201	extracellular matrix structural constituent	4/606	19/8384	0.043863102
GO:0003723	RNA binding	22/606	209/8384	0.047438785
GO:0008233	peptidase activity	35/606	363/8384	0.047690847
GO:0070011	peptidase activity, acting on L-amino acid peptides	34/606	352/8384	0.049405107
