# Supplementary material for: Comparative transcriptome analysis reveals the patterns of gene expression in different venison cuts of sika deer (Cervus nippon)
Source: Anim Biosci. 2025 May 12;38(11):2324–35. doi: 10.5713/ab.25.0044 (PMC12580950; doi:10.5713/ab.25.0044)
Supplement: Supplementary file 29 [file ab-25-0044-supplementary-29.pdf]

Supplement 29. The KEGG enrichment results of DEGs between BB and GM

KEGGID	Description	GeneRatio	BgRatio	pvalue
bta03008	Ribosome biogenesis in eukaryotes	18/379	86/8018	7.36E-08
bta04510	Focal adhesion	20/379	200/8018	0.001195128
bta04810	Regulation of actin cytoskeleton	21/379	234/8018	0.003465978
bta03013	Nucleocytoplasmic transport	13/379	120/8018	0.004244621
bta05218	Melanoma	9/379	71/8018	0.005944233
bta05135	Yersinia infection	14/379	141/8018	0.006678498
bta04010	MAPK signaling pathway	24/379	305/8018	0.009586818
bta04380	Osteoclast differentiation	12/379	118/8018	0.009671732
bta05207	Chemical carcinogenesis - receptor activation	16/379	185/8018	0.014051894
bta04650	Natural killer cell mediated cytotoxicity	10/379	97/8018	0.015935481
bta00513	Various types of N-glycan biosynthesis	6/379	45/8018	0.018344975
bta05223	Non-small cell lung cancer	9/379	88/8018	0.022677618
bta05212	Pancreatic cancer	8/379	77/8018	0.028316098
bta04664	Fc epsilon RI signaling pathway	7/379	64/8018	0.030547679
bta04217	Necroptosis	12/379	145/8018	0.041425264
bta05132	Salmonella infection	20/379	285/8018	0.049315856
