# Supplementary material for: Comparative transcriptome analysis reveals the patterns of gene expression in different venison cuts of sika deer (Cervus nippon)
Source: Anim Biosci. 2025 May 12;38(11):2324–35. doi: 10.5713/ab.25.0044 (PMC12580950; doi:10.5713/ab.25.0044)
Supplement: Supplementary file 30 [file ab-25-0044-supplementary-30.pdf]

Supplement 30. The GO enrichment results of DEGs between BB and GM

GOID	Description	GeneRatio	BgRatio	pvalue
GO:0006468	protein phosphorylation	47/244	456/5225	9.83E-08
GO:0016310	phosphorylation	48/244	485/5225	2.47E-07
GO:0051641	cellular localization	16/244	129/5225	0.000290922
GO:0046907	intracellular transport	14/244	108/5225	0.000441385
GO:0051649	establishment of localization in cell	14/244	109/5225	0.000485712
GO:0006913	nucleocytoplasmic transport	4/244	13/5225	0.002375278
GO:0051169	nuclear transport	4/244	13/5225	0.002375278
GO:0006886	intracellular protein transport	11/244	92/5225	0.003447248
GO:0034613	cellular protein localization	11/244	100/5225	0.006541531
GO:0070727	cellular macromolecule localization	11/244	100/5225	0.006541531
GO:0072594	establishment of protein localization to organelle	4/244	18/5225	0.008455557
GO:0015031	protein transport	11/244	108/5225	0.011486773
GO:0015833	peptide transport	11/244	108/5225	0.011486773
GO:0017038	protein import	3/244	11/5225	0.012557138
GO:0042886	amide transport	11/244	110/5225	0.013082616
GO:0045184	establishment of protein localization	11/244	110/5225	0.013082616
GO:0071705	nitrogen compound transport	12/244	127/5225	0.014906903
GO:0008104	protein localization	11/244	115/5225	0.017807401
GO:0033365	protein localization to organelle	4/244	23/5225	0.020384189
GO:0051056	regulation of small GTPase mediated signal transduction	9/244	90/5225	0.023731932
GO:0033036	macromolecule localization	13/244	154/5225	0.027016487
GO:1902531	regulation of intracellular signal transduction	9/244	96/5225	0.034281335
GO:0044431	Golgi apparatus part	4/137	29/3249	0.031676823
GO:0004672	protein kinase activity	48/434	461/8383	2.17E-06
GO:0004674	protein serine/threonine kinase activity	12/434	61/8383	5.47E-05
GO:0008536	Ran GTPase binding	4/434	13/8383	0.003485424
GO:0017016	Ras GTPase binding	10/434	88/8383	0.015128516
GO:0031267	small GTPase binding	10/434	88/8383	0.015128516
GO:0016765	transferase activity, transferring alkyl or aryl (other than methyl) groups	3/434	11/8383	0.016653748
GO:0016866	intramolecular transferase activity	4/434	22/8383	0.024672212
GO:0051020	GTPase binding	12/434	127/8383	0.031326743
GO:0005044	scavenger receptor activity	4/434	24/8383	0.033053307
GO:0038024	cargo receptor activity	4/434	24/8383	0.033053307
