# Supplementary material for: Comparative transcriptome analysis reveals the patterns of gene expression in different venison cuts of sika deer (Cervus nippon)
Source: Anim Biosci. 2025 May 12;38(11):2324–35. doi: 10.5713/ab.25.0044 (PMC12580950; doi:10.5713/ab.25.0044)
Supplement: Supplementary file 31 [file ab-25-0044-supplementary-31.pdf]

Supplement 31. The KEGG enrichment results of DEGs between T and GM

KEGGID	Description	GeneRatio	BgRatio	pvalue
bta00010	Glycolysis / Gluconeogenesis	19/828	74/8017	0.000130836
bta05162	Measles	28/828	134/8017	0.000199642
bta04120	Ubiquitin mediated proteolysis	31/828	164/8017	0.000602249
bta04630	JAK-STAT signaling pathway	25/828	130/8017	0.001531956
bta04066	HIF-1 signaling pathway	24/828	128/8017	0.002680066
bta03018	RNA degradation	18/828	87/8017	0.002951314
bta04213	Longevity regulating pathway - multiple species	15/828	68/8017	0.003376111
bta01522	Endocrine resistance	18/828	95/8017	0.007762802
bta00620	Pyruvate metabolism	11/828	48/8017	0.008437194
bta05206	MicroRNAs in cancer	29/828	178/8017	0.008532107
bta00051	Fructose and mannose metabolism	8/828	30/8017	0.009280034
bta04933	AGE-RAGE signaling pathway in diabetic complications	19/828	106/8017	0.011508442
bta04922	Glucagon signaling pathway	20/828	114/8017	0.012215011
bta04621	NOD-like receptor signaling pathway	26/828	161/8017	0.013742142
bta04915	Estrogen signaling pathway	22/828	131/8017	0.014559967
bta03050	Proteasome	11/828	52/8017	0.015431722
bta04340	Hedgehog signaling pathway	11/828	52/8017	0.015431722
bta04919	Thyroid hormone signaling pathway	22/828	132/8017	0.015830671
bta03083	Polycomb repressive complex	17/828	95/8017	0.016542909
bta04215	Apoptosis - multiple species	8/828	33/8017	0.016693699
bta04660	T cell receptor signaling pathway	20/828	120/8017	0.020767714
bta05214	Glioma	14/828	77/8017	0.024695854
bta04658	Th1 and Th2 cell differentiation	16/828	92/8017	0.025254398
bta04151	PI3K-Akt signaling pathway	49/828	360/8017	0.025592945
bta05230	Central carbon metabolism in cancer	15/828	85/8017	0.026336133
bta04625	C-type lectin receptor signaling pathway	17/828	100/8017	0.026390873
bta05220	Chronic myeloid leukemia	14/828	79/8017	0.030203827
bta04310	Wnt signaling pathway	26/828	173/8017	0.031693929
bta04380	Osteoclast differentiation	19/828	118/8017	0.032861506
bta05161	Hepatitis B	24/828	158/8017	0.033982508
bta04218	Cellular senescence	26/828	175/8017	0.03591581
bta05134	Legionellosis	12/828	66/8017	0.03593043
bta05222	Small cell lung cancer	16/828	96/8017	0.036059064
bta05169	Epstein-Barr virus infection	31/828	218/8017	0.040197386
bta04144	Endocytosis	36/828	261/8017	0.042676043
bta04970	Salivary secretion	15/828	92/8017	0.049024113
