# Supplementary material for: Comparative transcriptome analysis reveals the patterns of gene expression in different venison cuts of sika deer (Cervus nippon)
Source: Anim Biosci. 2025 May 12;38(11):2324–35. doi: 10.5713/ab.25.0044 (PMC12580950; doi:10.5713/ab.25.0044)
Supplement: Supplementary file 32 [file ab-25-0044-supplementary-32.pdf]

Supplement 32. The GO enrichment results of DEGs between T and GM

GOID	Description	GeneRatio	BgRatio	pvalue
GO:0006090	pyruvate metabolic process	8/542	11/5227	1.58E-06
GO:0006096	glycolytic process	8/542	11/5227	1.58E-06
GO:0006165	nucleoside diphosphate phosphorylation	8/542	11/5227	1.58E-06
GO:0006757	ATP generation from ADP	8/542	11/5227	1.58E-06
GO:0009132	nucleoside diphosphate metabolic process	8/542	11/5227	1.58E-06
GO:0009135	purine nucleoside diphosphate metabolic process	8/542	11/5227	1.58E-06
GO:0009179	purine ribonucleoside diphosphate metabolic process	8/542	11/5227	1.58E-06
GO:0009185	ribonucleoside diphosphate metabolic process	8/542	11/5227	1.58E-06
GO:0016052	carbohydrate catabolic process	8/542	11/5227	1.58E-06
GO:0042866	pyruvate biosynthetic process	8/542	11/5227	1.58E-06
GO:0046031	ADP metabolic process	8/542	11/5227	1.58E-06
GO:0046939	nucleotide phosphorylation	8/542	11/5227	1.58E-06
GO:0019359	nicotinamide nucleotide biosynthetic process	9/542	16/5227	7.62E-06
GO:0019363	pyridine nucleotide biosynthetic process	9/542	17/5227	1.47E-05
GO:0072525	pyridine-containing compound biosynthetic process	9/542	17/5227	1.47E-05
GO:0072330	monocarboxylic acid biosynthetic process	8/542	15/5227	4.22E-05
GO:0019362	pyridine nucleotide metabolic process	9/542	19/5227	4.62E-05
GO:0046496	nicotinamide nucleotide metabolic process	9/542	19/5227	4.62E-05
GO:0072524	pyridine-containing compound metabolic process	9/542	19/5227	4.62E-05
GO:0034655	nucleobase-containing compound catabolic process	11/542	28/5227	5.57E-05
GO:0009166	nucleotide catabolic process	8/542	16/5227	7.68E-05
GO:0006733	oxidoreduction coenzyme metabolic process	9/542	21/5227	0.000121342
GO:0044270	cellular nitrogen compound catabolic process	12/542	35/5227	0.00012143
GO:0046700	heterocycle catabolic process	12/542	35/5227	0.00012143
GO:0005975	carbohydrate metabolic process	26/542	118/5227	0.000135168
GO:1901292	nucleoside phosphate catabolic process	8/542	18/5227	0.000216233
GO:0019439	aromatic compound catabolic process	12/542	37/5227	0.000221955
GO:1901361	organic cyclic compound catabolic process	12/542	37/5227	0.000221955
GO:0032787	monocarboxylic acid metabolic process	9/542	23/5227	0.000278849
GO:0051186	cofactor metabolic process	15/542	55/5227	0.000328232
GO:0016053	organic acid biosynthetic process	8/542	19/5227	0.000339924
GO:0034404	nucleobase-containing small molecule biosynthetic process	8/542	19/5227	0.000339924
GO:0046394	carboxylic acid biosynthetic process	8/542	19/5227	0.000339924
GO:0016310	phosphorylation	73/542	482/5227	0.000375469
GO:0009152	purine ribonucleotide biosynthetic process	12/542	41/5227	0.000642111
GO:0009260	ribonucleotide biosynthetic process	12/542	41/5227	0.000642111
GO:0046390	ribose phosphate biosynthetic process	12/542	41/5227	0.000642111
GO:0044248	cellular catabolic process	24/542	116/5227	0.00065113
GO:0009108	coenzyme biosynthetic process	10/542	31/5227	0.000779301
GO:1901575	organic substance catabolic process	25/542	127/5227	0.001089728
GO:0006091	generation of precursor metabolites and energy	9/542	27/5227	0.001094111
GO:0006164	purine nucleotide biosynthetic process	12/542	44/5227	0.001282774

GO:0072522	purine-containing compound biosynthetic process	12/542	45/5227	0.001588247
GO:0046434	organophosphate catabolic process	8/542	24/5227	0.002069657
GO:0006732	coenzyme metabolic process	10/542	35/5227	0.002202574
GO:0009056	catabolic process	25/542	135/5227	0.002655071
GO:0006754	ATP biosynthetic process	8/542	25/5227	0.002772332
GO:0009142	nucleoside triphosphate biosynthetic process	8/542	25/5227	0.002772332
GO:0009145	purine nucleoside triphosphate biosynthetic process	8/542	25/5227	0.002772332
GO:0009201	ribonucleoside triphosphate biosynthetic process	8/542	25/5227	0.002772332
GO:0009206	purine ribonucleoside triphosphate biosynthetic process	8/542	25/5227	0.002772332
GO:0051188	cofactor biosynthetic process	10/542	37/5227	0.003455664
GO:0019318	hexose metabolic process	6/542	16/5227	0.00389786
GO:0009124	nucleoside monophosphate biosynthetic process	8/542	27/5227	0.004723446
GO:0009127	purine nucleoside monophosphate biosynthetic process	8/542	27/5227	0.004723446
GO:0009156	ribonucleoside monophosphate biosynthetic process	8/542	27/5227	0.004723446
GO:0009168	purine ribonucleoside monophosphate biosynthetic process	8/542	27/5227	0.004723446
GO:0006468	protein phosphorylation	64/542	453/5227	0.005041839
GO:0006913	nucleocytoplasmic transport	5/542	13/5227	0.007461435
GO:0051169	nuclear transport	5/542	13/5227	0.007461435
GO:0044283	small molecule biosynthetic process	10/542	42/5227	0.009093426
GO:0017144	drug metabolic process	12/542	55/5227	0.009269587
GO:0048583	regulation of response to stimulus	24/542	141/5227	0.009516163
GO:0048584	positive regulation of response to stimulus	5/542	14/5227	0.010633535
GO:0019752	carboxylic acid metabolic process	17/542	91/5227	0.011134212
GO:0009150	purine ribonucleotide metabolic process	12/542	57/5227	0.012344828
GO:0009259	ribonucleotide metabolic process	12/542	57/5227	0.012344828
GO:0006082	organic acid metabolic process	17/542	92/5227	0.012389839
GO:0043436	oxoacid metabolic process	17/542	92/5227	0.012389839
GO:0005996	monosaccharide metabolic process	6/542	20/5227	0.013171143
GO:0019693	ribose phosphate metabolic process	12/542	59/5227	0.016147654
GO:0009966	regulation of signal transduction	22/542	133/5227	0.017473493
GO:0010646	regulation of cell communication	22/542	133/5227	0.017473493
GO:0006163	purine nucleotide metabolic process	12/542	60/5227	0.018352122
GO:0023051	regulation of signaling	22/542	134/5227	0.018947659
GO:0035556	intracellular signal transduction	37/542	254/5227	0.019314494
GO:0072521	purine-containing compound metabolic process	12/542	61/5227	0.020774587
GO:0009165	nucleotide biosynthetic process	13/542	69/5227	0.023137342
GO:1901293	nucleoside phosphate biosynthetic process	13/542	69/5227	0.023137342
GO:0048519	negative regulation of biological process	12/542	62/5227	0.023426787
GO:0006511	ubiquitin-dependent protein catabolic process	9/542	43/5227	0.029428663
GO:0019941	modification-dependent protein catabolic process	9/542	43/5227	0.029428663
GO:0043632	modification-dependent macromolecule catabolic process	9/542	43/5227	0.029428663
GO:0032268	regulation of cellular protein metabolic process	5/542	18/5227	0.032150846
GO:0009057	macromolecule catabolic process	13/542	74/5227	0.038923273
GO:0051246	regulation of protein metabolic process	5/542	19/5227	0.040032195

GO:0044265	cellular macromolecule catabolic process	12/542	67/5227	0.040520519
GO:0046034	ATP metabolic process	8/542	39/5227	0.043392615
GO:0009144	purine nucleoside triphosphate metabolic process	8/542	40/5227	0.049529296
GO:0009199	ribonucleoside triphosphate metabolic process	8/542	40/5227	0.049529296
GO:0009205	purine ribonucleoside triphosphate metabolic process	8/542	40/5227	0.049529296
GO:0005576	extracellular region	31/266	217/3243	0.001235469
GO:0005654	nucleoplasm	11/266	63/3243	0.012049741
GO:0044451	nucleoplasm part	11/266	63/3243	0.012049741
GO:0016591	DNA-directed RNA polymerase II, holoenzyme	4/266	12/3243	0.01291703
GO:0000428	DNA-directed RNA polymerase complex	4/266	14/3243	0.022925252
GO:0055029	nuclear DNA-directed RNA polymerase complex	4/266	14/3243	0.022925252
GO:0031012	extracellular matrix	4/266	16/3243	0.036613412
GO:0031981	nuclear lumen	12/266	85/3243	0.042387793
GO:0030133	transport vesicle	3/266	10/3243	0.042461722
GO:0031248	protein acetyltransferase complex	3/266	10/3243	0.042461722
GO:1902493	acetyltransferase complex	3/266	10/3243	0.042461722
GO:0016830	carbon-carbon lyase activity	8/936	20/8372	0.000869678
GO:0016831	carboxy-lyase activity	7/936	17/8372	0.001509779
GO:0016829	lyase activity	14/936	57/8372	0.003315742
GO:0004672	protein kinase activity	67/936	458/8372	0.011774203
GO:0004674	protein serine/threonine kinase activity	13/936	61/8372	0.015655411
GO:0003723	RNA binding	34/936	210/8372	0.016438092
GO:0060589	nucleoside-triphosphatase regulator activity	12/936	55/8372	0.016553669
GO:0030695	GTPase regulator activity	11/936	49/8372	0.017340016
GO:0030234	enzyme regulator activity	24/936	139/8372	0.019664685
GO:0005262	calcium channel activity	4/936	11/8372	0.02690218
GO:0008081	phosphoric diester hydrolase activity	10/936	47/8372	0.032226897
GO:0005520	insulin-like growth factor binding	5/936	17/8372	0.033991568
GO:0019842	vitamin binding	9/936	42/8372	0.039402996
GO:0098772	molecular function regulator	53/936	376/8372	0.04307131
GO:0005044	scavenger receptor activity	6/936	24/8372	0.04436769
GO:0038024	cargo receptor activity	6/936	24/8372	0.04436769
GO:0004435	phosphatidylinositol phospholipase C activity	4/936	13/8372	0.048666414
GO:0004629	phospholipase C activity	4/936	13/8372	0.048666414
GO:0008134	transcription factor binding	4/936	13/8372	0.048666414
